# Congenital hypoplasia of the cerebellum: developmental causes and behavioral consequences

**DOI:** 10.3389/fnana.2013.00029

**Published:** 2013-09-03

**Authors:** M. Albert Basson, Richard J. Wingate

**Affiliations:** ^1^Department of Craniofacial Development and Stem Cell Biology, King’s College LondonLondon, UK; ^2^Medical Research Council Centre for Developmental Neurobiology, King’s College LondonLondon, UK

**Keywords:** cerebellum, development, defects, hypoplasia, genetics, function, behavior, autism spectrum disorders

## Abstract

Over the last 60 years, the spotlight of research has periodically returned to the cerebellum as new techniques and insights have emerged. Because of its simple homogeneous structure, limited diversity of cell types and characteristic behavioral pathologies, the cerebellum is a natural home for studies of cell specification, patterning, and neuronal migration. However, recent evidence has extended the traditional range of perceived cerebellar function to include modulation of cognitive processes and implicated cerebellar hypoplasia and Purkinje neuron hypo-cellularity with autistic spectrum disorder. In the light of this emerging frontier, we review the key stages and genetic mechanisms behind cerebellum development. In particular, we discuss the role of the midbrain hindbrain isthmic organizer in the development of the cerebellar vermis and the specification and differentiation of Purkinje cells and granule neurons. These developmental processes are then considered in relation to recent insights into selected human developmental cerebellar defects: Joubert syndrome, Dandy–Walker malformation, and pontocerebellar hypoplasia. Finally, we review current research that opens up the possibility of using the mouse as a genetic model to study the role of the cerebellum in cognitive function.

## INTRODUCTION

The cerebellum is an intriguing component of the central nervous system. From one perspective it is a famously simple neuronanatomical circuit constructed from a relatively few neuronal types and comprising a single uniform microarchitecture (Cajal, 1894; Eccles et al., 1967). However, the nature of the calculations performed by this circuit and its precise role in a variety of different neural functions has proved notoriously difficult to pin down. Despite the conserved nature of its core functional neuronal partnership, formed between granule cell axons and Purkinje cell dendrites, it is also clear that the cerebellum is employed as a neural “comparator” in different ways in different species (Meek, 1992; Barlow, 2002). From a predominantly proprioceptive and sensory role in fish, it has adopted more overt motor functions in mammals (Nieuwenhuys et al., 1998). In primates, including humans, a large proportion of the cerebellar cortex is in addition given over to interactions with regions of the cortex involved in cognition and judgment (Strick et al., 2009). The recruitment of a relatively unchanging core cerebellar circuitry into a variety of different functions both presents challenges in understanding its role in human disease but also great potential for the use of simpler model animal systems in solving these challenges.

Despite the uniformity of its cellular structure, the cerebellum is divided into clear anatomical divisions on the basis of a transverse fissures that separate lobes. These are folds in what is a continuous ribbon of neural circuitry that, in humans, would extend over a meter in anteroposterior length (Braitenberg and Atwood, 1958). A primary fissure divides the anterior from the posterior lobes, while a posterolateral fissure separates posterior lobe from a distinct flocculonodular lobe. Perpendicular to these, longitudinal, deep furrows partition the two cerebellar hemispheres (both with intermediate and lateral zones) from a central “vermis.” While the flocculonodular lobe sits somewhat apart as a region with direct vestibular interactions (the “vestibulocerebellum”), the vermis, intermediate and lateral cerebellar hemispheres each predominantly target a different cerebellar nucleus that lies in the white matter beneath the cerebellar cortical layers. Thus for the majority for the cerebellum, the targeting of output of each nucleus determines the functional output of the overlying cerebellar cortex. In mammals, the medial vestigial and interposed nuclei mainly target descending motor systems, channeling the output of the vermis and intermediate hemispheric zone (the “spinocerebellum”). By contrast, the lateral zone of the cerebellar hemispheres is chiefly linked via the dentate nucleus to the thalamus and hence the cerebral cortex (the “cerebrocerebellum”).

These three major functional subdivisions of the cerebellum have been long been recognized and used to calibrate defects in developmental morphogenesis, many of which, as described below, have a prominent affect on the vermis (spinocerebellum). However, it should be clear from the above that functional consequences of developmental disorders may also depend on the degree of disruption to the formation of cerebellar nuclei and the precision of their inputs, which are difficult to assess. In addition, appreciation of cerebellar dysfunction is colored by the simple constraint that the cerebellum is best-known and understood in terms of its integration of proprioceptive information in the control of movement (Sherrington, 1906; Holmes, 1939). Typical symptoms of cerebellar dysfunction include dyssynergia (problems with measuring appropriate muscle force), dysmetria (improper interpretation of distance), ataxia (disordered movement), and dysdiadochokinesia (inability to perform rapidly alternating movements). Therefore, although appreciated for some time, relatively little attention has been given to the involvement of the cerebellar system in cognitive and emotional behaviors. This might reflect both the immediate usefulness of simple motor tests to diagnose cerebellar damage (Holmes, 1939), but also a focus on descending motor systems that corresponds to the expectations of loss of the vermis.

However, recent studies have highlighted the possibility that cerebellar defects might underlie some of the symptoms in subsets of patients diagnosed with neurodevelopmental disorders like autism spectrum disorders (ASD), attention deficit hyperactivity disorder (ADHD), and schizophrenia (Courchesne et al., 1988; Bauman and Kemper, 1994; Mostofsky et al., 1998; Palmen et al., 2004; Bottmer et al., 2005; Fatemi et al., 2012; Vaidya, 2012; Villanueva, 2012). As these conditions are clinically heterogeneous, it remains near impossible to consistently link specific neuro-anatomical defects, in the cerebellum or otherwise, to distinct behaviors. However, as our ability to classify patients into more homogeneous phenotypic groups improves with ever more powerful imaging techniques (Smyser et al., 2011; Haubold et al., 2012), and next generation sequencing approaches allows the identification of genetic alterations (Coe et al., 2012), the possibilities of linking genetic alterations with specific cerebellar defects and behaviors becomes imminently feasible. These analyses extend to conditions characterized by vermal agenesis, emphasizing the need for a fuller understanding of how cerebellar output and underlying anatomy are reorganized in these conditions.

These advances are coupled with an increasing potential to interpret both anatomical and genetic phenotypes in terms of specific aspects of cerebellar development. This has been driven by substantial progress in understanding the origins of different cerebellar cell types and their interactions within the last 10 years. Fate-maps based on the genetic identity of different cell types, a molecular dissection of their interactions and new anatomical techniques to trace long range connections in the brain (Strick et al., 2009) have revealed the underlying pathways for cerebellum growth and patterning. In particular, distinct developmental pathways for neurons with the cerebellar cortex and deep nuclei imply that different populations will be affected in different ways depending on the location and timing of a given genetic disorder. These point the way to a future where specific connections and neuron populations can be systematically investigated in the context of human disorders. However, our understanding of the mechanisms whereby these genetic alterations cause specific cerebellar pathologies and the exact behavioral consequences of these cerebellar defects remain limited. These questions are best addressed in model systems that allow the accurate perturbation of specific genes and/or pathways, coupled with an in-depth analysis of developmental processes over time. The mouse has emerged as a valuable model for three critical regions: (1) powerful genetic tools available in the mouse have made it possible to accurately fate-map cells that share the same genetic ancestry and (2) dissect the function of a gene at different developmental time points and in different cell types or brain regions with high precision, and (3) behavioral tests have been developed that can be applied to determine the consequences of defined defects on specific behavioral endo-phenotypes. The development of innovative approaches to map brain connectivity in mice will add yet another powerful tool to the available kit (Lo and Anderson, 2011).

As the classification of brain pathologies with cerebellar involvement and known genetic associations have been reviewed extensively (see for example Barkovich et al., 2009), our aim is not to recapitulate these in the present article. Instead, we aim to outline some of the key developmental processes that typically go awry during cerebellar development and use well-understood examples from mouse genetic studies to illustrate how different developmental defects or signaling defects that arise at different developmental stages cause distinct structural abnormalities of the cerebellum. This discussion highlights significant gaps in our understanding of the mechanisms that underlie cerebellar malformation. Finally, we briefly outline the current understanding of cerebellar connectivity with the neocortex that might underlie its role in higher order function and speculate how defects in cerebellar connectivity might underlie behaviors associated with neurodevelopmental disorders such as autism. This final section highlights the potential limits of the mouse model as a means of understanding the full range of cerebellar developmental disorders, when the functional connections between cerebellum, thalamus, and cortex are not yet fully understood.

## CONGENITAL CEREBELLAR DEFECTS: DEVELOPMENTAL MECHANISMS

Developmental defects of the cerebellum can be present as part of more complex developmental syndromes, in combination with other nervous system defects such as cortical hypoplasia and corpus callosum agenesis or more rarely, as isolated defects. Clinical classification of cerebellar defects is difficult and several classification schemes have been proposed, some of which are based on embryological and genetic considerations (Barkovich et al., 2009). These classifications are important in order for the correct division of patients for treatment and further genetic studies to identify the genetic causes responsible for cerebellar anomalies. Before we discuss recent advances in the genetics of human cerebellar hypoplasia, we first provide an overview of the key developmental processes that control cerebellar growth and morphogenesis, and discuss pertinent studies in mouse mutants upon which most of the interpretation of human malformations is based.

### OVERVIEW OF KEY STAGES IN CEREBELLAR DEVELOPMENT: INSIGHTS FROM THE MOUSE

#### The isthmus organizer (IsO)

**FIGURE 1:**
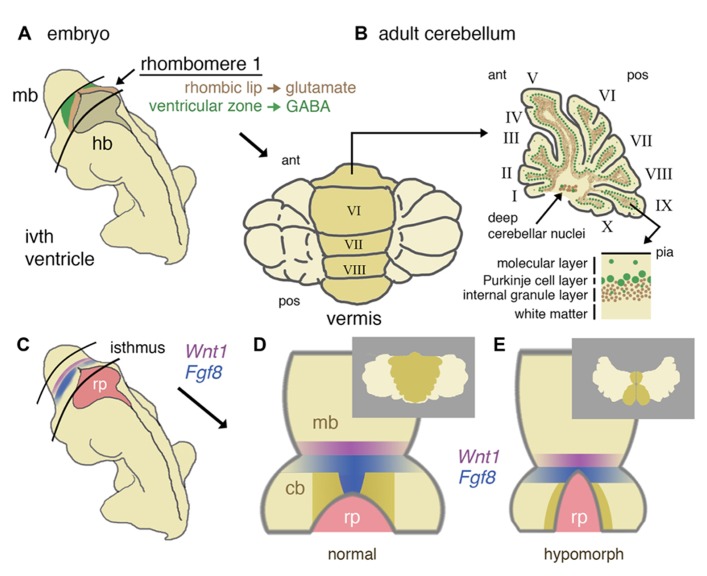
**Developmental origins of the mouse cerebellum and the role of isthmic gene expression in patterning the vermis. (A)** Schematic representation of a mid-gestation embryo showing the location of derivatives of rhombomere 1. The ventricular layer (green) and rhombic lip (brown) of dorsal rhombomere 1 give rise to all GABA-ergic and glutamatergic cells of the cerebellum, respectively. **(B)** In a dorsal (posterior) view, the adult cerebellum is characterized by a central (darker shaded) vermis running anterior (ant) to posterior (pos). A uniform layering of cell types can be found throughout the vermis and more lateral hemispheres (shown in schematic parasagittal section), with GABA-ergic and glutamatergic differentially distributed in a later-specific manner: the molecular layer is largely reserved for the interaction of Purkinje cell dendrites and granule cell axons with sparse basket and stellate inhibitory interneurons. The Purkinje cells layer separates the molecular layer from an internal granule cell layer that contains a population of inhibitory Golgi cells. Deep cerebellar nuclei (GABA-ergic and glutamatergic neurons) lie within the white matter. **(C)** Schematic diagram showing the location of the isthmus organizer at the midbrain/hindbrain boundary with respect to the fourth ventricle roof plate (rp) and the expression domains of *Wnt1* (purple) and *Fgf8* (blue). **(D)** Dorsal schematic view of the isthmus region showing with darker shading the approximate region where progenitors of the cerebellar vermis reside, as based on inducible fate-mapping studies (Sgaier et al., 2005). The translation of this dorsal rhombomere 1 territory into adult vermis is shown **inset**. **(E)** Altered morphology of the isthmic region and reduced cerebellar size in a hypomorph with an altered function of the isthmic organizer due to diminished FGF signaling. Loss of vermis progenitors is concomitant with the expansion of the roof plate (adapted from Basson et al., 2008). The consequences for vermal morphogenesis in the adult are shown **inset**.

The cerebellum is derived from the dorsal part of the most anterior segment of the hindbrain, rhombomere 1 (r1; Millet et al., 1996; Wingate and Hatten, 1999). Thus, any developmental defect that results in the failure to specify the anterior hindbrain or r1 itself, will inevitably result in cerebellar aplasia (Eddison et al., 2004) as might global defects in dorsal patterning mechanisms (Chizhikov et al., 2006). Early steps in brain development include the specification of neural tissue (neural induction), formation, and internalization of the neural tube (neurulation), and patterning of the neural tube. The latter process imparts positional identity to different compartments along the anterior–posterior neuraxis, a process primarily achieved through the formation of specialized signaling centers, also referred to as secondary organizers. Secondary organizers secrete growth factors that pattern the adjacent tissue through the induction of distinct patterns of gene expression on either side of the organizer, as a result of the presence of the differential expression of competence factors (Kiecker and Lumsden, 2012). The signaling center that divides and patterns the mesencephalon and r1 is the mid-hindbrain or IsO. Classic studies in a number of model organisms have shown that the key organizing molecule secreted by the IsO is fibroblast growth factor 8 (FGF8; Crossley et al., 1996; Martinez et al., 1999). The initiation of *Fgf8* expression at the IsO is dependent upon the transcription factor LMX1B (Lim homeobox transcription factor 1 beta), whereas the position of the IsO at the mid-hindbrain boundary is determined the mutually repressive activities of the homeobox genes *Otx2* (orthodenticle homeobox 2) anteriorly, and *Gbx2* (gastrulation brain homeobox 2), posteriorly (Joyner et al., 2000; Guo et al., 2007). Once established, a stable transcriptional and signaling network maintains gene expression at the IsO. Critical components of this regulatory network include the transcription factors PAX2 (paired box gene 2), EN1 (engrailed 1), EN2 (engrailed 2), and GLI3 (GLI-Kruppel family member 3) and signaling molecules FGF8, FGF17, WNT1 (wingless-type MMTV integration site family, member 1), and SHH (Sonic Hedgehog; Wittmann et al., 2009). Detailed fate-mapping studies in the mouse have located the progenitors of the medial cerebellar vermis to anterior r1 of the early embryo (Sgaier et al., 2005). The spatial organization of gene expression patterns of *Wnt1* and *Fgf8* in relation to the approximate progenitor domains of the vermis and hemispheres are represented in **Figure 1**.

Conditional gene deletion experiments in the mouse have proven to be an extremely powerful approach to dissect different requirements of key signaling pathways during cerebellar development (**Table 1**). The FGF and WNT signaling pathways are prime examples. Since the initial identification of *Fgf8* and *Wnt1* gene expression in cells at the IsO (Wilkinson et al., 1987; Crossley and Martin, 1995), various approaches to disrupt the function of these genes during cerebellar development have been employed. The germline deletion of *Fgf8* revealed an early function in gastrulation, such that the role of *Fgf8* in cerebellar development could not be investigated in these mutants (Meyers et al., 1998). The deletion of *Fgf8* specifically from the early IsO was found to result in the rapid cell death of all progenitors of the midbrain and cerebellum, identifying FGF as an essential survival factor cells in the mesencephalic(mes)/r1 region. The analysis of embryos homozygous for hypomorphic alleles of *Fgf8*, suggested that the maintenance of normal levels of FGF8 signaling was particularly important for the formation of medial cerebellar tissue (Chi et al., 2003). The requirement for high FGF signaling during vermis development was confirmed in mouse mutants where FGF signaling was specifically inhibited in the developing mes/r1 region shortly after the initiation of *Fgf8* expression in the IsO. Furthermore, the loss of vermis progenitors was found to be associated with roof plate expansion in anterior r1 (Basson et al., 2008; **Figures 1D, E**). A study by the Joyner lab has shown that the developmental stage at which *Fgf8* expression is disrupted is a key determinant of the severity of vermis hypoplasia; *Fgf8 *deletion from the early (pre-E9.5) IsO cause severe vermis hypoplasia, whereas only mild hypoplasia in the anterior vermis resulted from *Fgf8* deletion between E9.5 and E11 (Sato and Joyner, 2009). 

**Table 1:** Examples of mouse models that have informative cerebellar phenotypes and characterized developmental defects.

Mouse model	Developmental defects	Cerebellar phenotype	Comments
*En1*^cre/^^+^;Fgf8^flox/flox^ (conditional *Fgf8* deletion in mes/r1)	Programmed cell death of mes/r1 progenitors (Chi etal., 2003)	Agenesis	Germline *Fgf8* loss or hypomorphic mutations are embryonic lethal.
*En1*^cre/^^+^;Fgfr1^flox/flox^ (conditional *Fgfr1* deletion in mes/r1)	Defect in specification and/or expansion of vermis progenitors (Trokovic etal., 2003)	Vermis a/hypoplasia	Conditional gene deletion approaches have revealed different temporal and quantitative requirements for FGF signaling in vermis vs. hemisphere formation.
*En1*^cre/^^+^;Spry2GOF;Fgf8+/- (reduction of FGF signaling specifically in the mes/r1 region)	Cell death in mes, failure to specify inferior colliculus (IC), defect in specification and/or expansion of vermis progenitors, roof plate expansion (Basson etal., 2008)	Vermis a/hypoplasia	
*Wnt1-/-*	Loss of mes/r1 progenitors (McMahon and Bradley, 1990)	Agenesis	Vermis and hemispheres have different quantitative and temporal requirements for WNT signaling, similar to FGF
*Wnt1*^sw/sw^ (*Wnt1* hypomorph)	Defect in specification and/or expansion of vermis progenitors, roof plate expansion (Thomas etal., 1991; Louvi etal., 2003)	Vermis a/hypoplasia	
*Ahi1-/-*	Expanded roof plate; reduced WNT signaling and progenitor expansion in the medial vermis (Lancaster etal., 2009)	Vermis hypoplasia	*AHI1* mutations cause Joubert syndrome (Dixon-Salazar etal., 2004; Ferland etal., 2004)
*Tmem67-/-*	Expanded roof plate	Vermis hypoplasia	*TMEM67* (*MKS3*) mutations cause Joubert syndrome (Baala etal., 2007)
*En1-/-*	Failure to specify IC, defect in specification and/or expansion of vermis progenitors (Wurst etal., 1994)	Vermis a/hypoplasia	
*En2-/-*	Altered cerebellar foliation and growth. *En2* controls timing of fissure formation together with *En1* (Cheng etal., 2010; Orvis etal., 2012)	Subtle foliation defects (Joyner etal., 1991)	*EN2* polymorphisms linked to autism (Gharani etal., 2004)
*Gbx2-/-*	Failure of r1 specification and transformation into mes (Millet etal., 1999)	Cerebellar agenesis	No human mutations identified yet
*Gbx2*^neo/neo^ (Gbx2 hypomorph)	Defects in anterior r1 (Waters and Lewandoski, 2006)	Vermis hypoplasia	
*En1*^Otx2/^^+^**(misexpression of *Otx2* through the mes/r1 region)	Caudal repositioning of IsO, expansion of mesencephalon at the expense of anterior r1 (vermis progenitors; Broccoli etal., 1999)	Vermis aplasia	
*Lmx1b-/-*	Defect in establishing and maintaining the IsO (Guo etal., 2007)	Vermis agenesis	
*Lmx1a-/-*	Smaller roof plate	Mild cerebellar hypoplasia	
*Lmx1a-/-;Lmx1b*^cko/^^-^	Very small roof plate (Mishima etal., 2009)	Severe cerebellar hypoplasia	
*Ptf1a-/-*	Ventricular zone defects (Hoshino etal., 2005; Pascual etal., 2007)	Severe cerebellar hypoplasia	Cerebellar agenesis in humans with homozygous *PTF1A* loss of function (Sellick etal., 2004)
*Atoh1-/-*	Failure to form EGL (Ben-Arie etal., 1997)	Severe cerebellar hypoplasia	*Atoh1* is required for *Shh*-responsiveness and GCp proliferation (Flora etal., 2009)
*L7-Cre;Shh*^flox/flox^ (conditional deletion of *Shh* from PCs)	Reduced GCp proliferation (Lewis etal., 2004)	General cerebellar hypoplasia	
*hGFAP-Cre;Smo*^flox/flox^ hGFAP-Cre;Kif3a^flox/flox^ Conditional deletion of *Smo* and *Kif3a* from GCp lineage	Reduced GCp proliferation (Spassky etal., 2008)	General cerebellar hypoplasia	Similar phenotypes have been reported upon deletion of other downstream effectors of SHH signaling, e.g., *Gli2*******(Corrales etal., 2004, 2006)
*Zic1-/-;Zic4-/-*	Reduced GCp proliferation, unknown developmental causes for anterior vermis defects (Blank etal., 2011)	Cerebellar hypoplasia	*ZIC1+/-;ZIC4+/-* haploinsufficiency linked to Dandy–Walker malformation (Grinberg etal., 2004; Tohyama etal., 2011)
*Foxc1-/-*	Enlarged roof plate, disorganized rhombic lip, loss of Atoh1 expression in medial cerebellum, reduced *Tgfb1*, *Cxcl12*, *Bmp2****,*** and *Bmp4* expression (Aldinger etal., 2009)	Vermis a/hypoplasia	*FOXC1* deletions or duplications associated with cerebellar hypoplasia and Dandy–Walker malformation in humans (Aldinger etal., 2009)
*Pcp2-cre;Tsc1*^flox/flox^ (Purkinje cell-specific deletion of *Tsc1*)	Apparent normal cerebellar development (Tsai etal., 2012)	Postnatal Purkinje cell loss and increased dendritic spine density (Tsai etal., 2012)	*TSC1* or *TSC2* mutations associated with cerebellar tubers (tuberous sclerosis) and autism (Eluvathingal etal., 2006)
*Reln*	Defects in PC migration and secondary GCp expansion (Mariani, 1982; Miyata etal., 2010)	Severe cerebellar hypoplasia (Magdaleno etal., 2002)	*RELN-/-* patients have cerebellar hypoplasia (Hong etal., 2000)
*Vldlr-/-* and *Apoer2-/-*(genes encoding Reln receptors)	Abnormal PC migration and dendritic arborization (Trommsdorff etal., 1999)	General cerebellar hypoplasia	*VLDLR-/-* patients have pontocerebellar hypoplasia and quadrupedal locomotion (Ozcelik etal., 2008)

In the case of *Wnt1*, the germline deletion of *Wnt1* resulted in a similar phenotype to the early mes/r1-deletion of *Fgf8*, namely the absence of the midbrain and cerebellum by birth (McMahon and Bradley, 1990; Thomas and Capecchi, 1990). A similar phenotype is observed upon the deletion of β-catenin using a *Wnt1-Cre* line (Brault et al., 2001). The cerebella of mice homozygous for a hypomorphic allele of *Wnt1* (*swaying, sw*) essentially represent phenocopies of FGF hypomorphic cerebella, by displaying a specific loss of the cerebellar vermis (Thomas et al., 1991; Louvi et al., 2003). Temporal requirements for WNT signaling have not been mapped as extensively as for FGF, but the deletion of β-catenin after E12.5 using Nestin-Cre, resulted in cerebellar vermis hypoplasia defects similar to *Wnt1*^sw/sw^mutants (Schuller and Rowitch, 2007). This observation suggests that the requirement for WNT/β-catenin signaling during vermis development and midline “fusion” is later, or extends over a longer time window than the requirement for FGF signaling. Taken together, these studies indicate that the cerebellar vermis that develops from tissue in anterior r1 that is exposed to the highest levels of FGF and WNT for the longest time has the strictest requirement for these signals during development.

In keeping with this general theme, mice deficient in *En1*, the first engrailed homeobox gene to be expressed during cerebellar development, results in cerebellar vermis aplasia (Wurst et al., 1994). A substantial number of mice with conditional deletion of *En1* after E9 exhibit normal cerebella, confirming the importance of early *En1* expression (Sgaier et al., 2007).

The role of the SHH pathway in postnatal cerebellar development is well-understood (see below). However, recent studies have provided evidence for important roles for *Gli3* at the IsO, consistent with *Gli3* as a regulator of dorsal neural tube cell fates. Deletion of *Gli3 *results in higher *Fgf8* expression and the expansion of the IsO (Aoto et al., 2002; Blaess et al., 2008). Recently, the conditional deletion of the SHH regulator, SUFU (suppressor of fused homolog), was shown to cause hyperplasia and disorganization of the IsO and mes/r1 regions. These early defects were also associated with ectopic and disorganized *Fgf8* expression at the IsO. Interestingly, these SUFU mutants exhibited cerebellar vermis hypoplasia that was almost entirely rescued by the constitutive expression of the GLI3 repressor (GLI3R) form, suggesting that this defect was primarily caused by the failure to generate GLI3R (Kim et al., 2011).

An important prediction of these studies in the mouse is that many human cerebellar disorders with strong cerebellar vermis involvement are likely to be caused by the disruption of IsO function during the earliest stages of cerebellar development. This proposition will be discussed further in Section “Mechanistic Insights into the Causes of Human Cerebellar Defects” in the context of recent findings on the mechanisms underlying human cerebellar malformations.

#### Establishment of progenitor zones and neurogenesis

**FIGURE 2:**
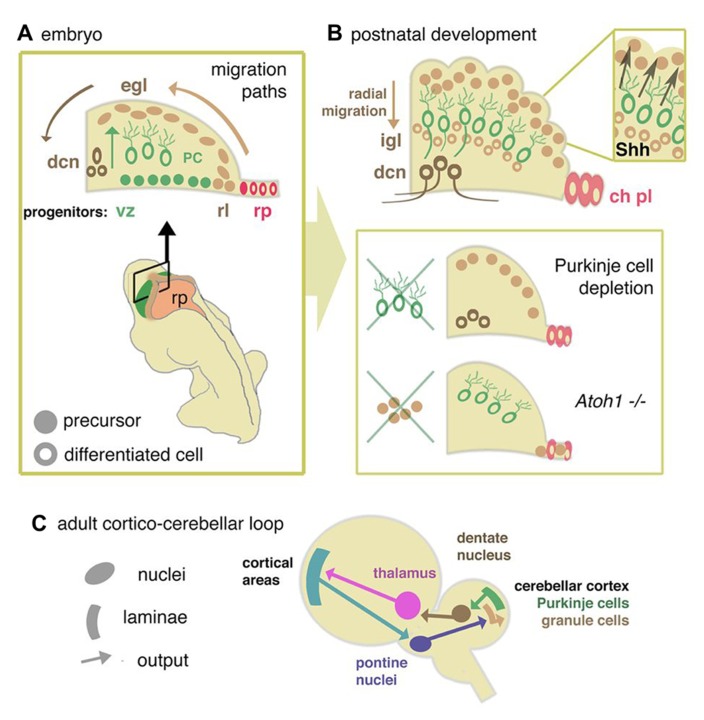
**Cellular development of cerebellum and components of the re-entrant cortico-cerebellar loop. (A)** Schematic cross sectional view through the anlage of the cerebellum showing the relationship between rhombic lip (rl), ventricular zone (vz), and roof plate (rp) of the fourth ventricle. Adult morphological layering (Figure1) is the product of two major migration pathways. The vz gives rise to GABA-ergic radially dispersed Purkinje cells (**oc**). The rl generates glutamatergic deep cerebellar nucleus (**dcn**) neurons and granule cell precursors of the external germinal layer (**egl**), which migrate tangentially from the rl in sub-pial streams. **(B)** Postnatally, the egl proliferates under the influence of Purkinje cell-derived Shh. Postmitotic glutamatergic granule cells migrate radially from the EGL to internal granule layer (**igl**). In mutants where Purkinje cells are deleted, or in which Shh is depleted, or where egl formation is suppressed (as in the *Atoh1* knockout mouse), cerebellum growth is reduced. Disruptions in signals from the overlying mesenchyme, directly or through affecting signaling from the roof plate, may modulate the responsiveness of the egl to mitogens, thus abrogating its expansion. **(C)** From data derived in the primate (Strick et al., 2009) a general model for a mammalian circuit would propose that cortico-cerebellar closed loops modulate cortical activity for a number of different motor and non-motor cortical areas. Without presupposing details of cortical areas in the mouse, cortical activity would be anticipated to feed into the cerebellar circuit via the pontine nucleus, a derivative of the rhombic lip of the hindbrain. Cerebellar output via dentate nucleus neurons would then feed back to the cortical areas via the thalamus.

After initial patterning and growth of r1 to form the cerebellar anlage, neurogenesis is initiated in two distinct germinal centers, the ventricular zone (VZ) and rhombic lip (RL). All cerebellar neurons and glia as well as progenitors that populate a number of extracerebellar nuclei are born within these germinal zones (**Figures 1A, B** and **Figure 2A**). Evidence that the production of different neuronal lineages is spatially restricted during cerebellar development comes from loss-of-function and lineage tracing experiments in the mouse. Ben-Arie et al. (1997) first demonstrated that the loss of *Atoh1* (atonal homolog 1), a gene specifically expressed in the RL, resulted in the failure to form an external germinal layer (EGL) and EGL-derived granule cells (**Figure 2B**).

***The rhombic lip***. Genetic fate-mapping studies and *Atoh1* loss-of-function studies have shown that progenitors of all excitatory glutamatergic neurons of the cerebellum are generated within the upper RL (Machold and Fishell, 2005; Wang et al., 2005). Defects in the formation or induction of the RL or the specification of granule cell progenitors (GCps) are predicted to result in severe cerebellar hypoplasia due to the absence of this rapidly proliferating transit amplifying cell population during postnatal development (see Progenitor Cell Migration, Proliferation, and Differentiation). The mechanisms required for the induction and functionality of the RL are being elucidated. A number of signaling pathways, including the TGFβ (transforming growth factor beta) and Notch pathways and signaling from the roof plate (Alder et al., 1996; Chizhikov et al., 2006) are implicated in the induction of the RL. Cell production from the RL appears to involve an iterative induction of *Atoh1* in successive waves of migratory derivatives (Machold et al., 2007; Broom et al., 2012).

In addition to GCps, the *Atoh1*-positive RL also gives rise to neurons that populate the deep cerebellar and extracerebellar nuclei; these include both glutamatergic and cholinergic neurons (Machold and Fishell, 2005; Wang et al., 2005; Wingate, 2005). Moreover, the RL extends into the hindbrain where it generates neurons that participate in a number of defined circuits including mossy fiber inputs to granule cells via the pons (Rodriguez and Dymecki, 2000; Rose et al., 2009). This raises the possibility that defects across the extent of the cerebellar and hindbrain RL could be the cause of conditions, such as pontocerebellar hypoplasia (see below) where multiple distributed elements of the cerebellar systems are disrupted. Developmental defects affecting this progenitor zone and its descendants might have far-reaching effects on cerebellar connectivity (**Figure 2A**). In particular, these discoveries also point to a time window of sensitivity to developmental damage that might target deep cerebellar nuclei but leave later born granule cell derivatives untouched. Such a window of potential vulnerability to intrinsic or extrinsic damage to the embryo might have selective effects on cerebellar function (in particular connectivity) that are not necessarily correlated with substantial reduction in cerebellar size.

***Ventricular zone***. Genetic fate-mapping of cells in the cerebellar VZ, demonstrated that all GABA-ergic neurons, including Purkinje, Golgi, basket, and stellate cells, as well as small GABA-ergic neurons of the deep cerebellar nuclei are derived from this region (Hoshino et al., 2005; Sudarov et al., 2011; **Figure 2A**). Compared to the number of glutamatergic granule neurons in the adult cerebellum, the contribution of GABA-ergic neurons to the over-all size of the cerebellum is relatively minor. Thus, defects in the generation of GABA-ergic neurons are not expected to result directly in significant cerebellar hypoplasia. However, as we discuss in the next section, VZ-derived Purkinje cell progenitors are the primary source of mitogen to GCps in the EGL. Thus, the absence or mislocalization of Purkinje cells due to VZ defects could be responsible for cerebellar hypoplasia owing to a deficit in GCp proliferation and postnatal cerebellar growth (**Figure 2B**). Indeed, Hoshino et al. (2005) showed that the disruption of the Ptf1a (pancreas transcription factor 1 subunit alpha) gene by transgenic insertion resulted in the compete loss of GABA-ergic lineages in the cerebellum and severe cerebellar hypoplasia. It is important to note that Bergmann glia are also derived from the VZ. As these cells form the scaffold that guides the radial migration of neuronal progenitors, defects in the generation or differentiation of these cells could also be responsible for the failure of Purkinje cell migration (see Progenitor Cell Migration, Proliferation, and Differentiation).

Finally, evidence for interaction between progenitor zones comes from the analysis of cell fate upon the deletion of *Ptf1a* and *Atoh1*. Pascual et al. (2007) showed that VZ-derived progenitors that develop in the absence of the transcription factor PTF1A, invade the EGL and adapted glutamatergic fates reminiscent of RL-derived progenitors, indicating that PTF1A actively represses glutamatergic fate to maintain GABA-ergic fate determination. Similarly, Rose et al. (2009) found that deletion of *Atoh1* results in RL cells entering the roof plate, indicating that ATOH1 activity suppresses the adoption of this non-neuronal fate. This is reminiscent of the mutual inhibitory interactions that specify progenitor domains within the spinal cord (reviewed by Wilson and Maden, 2005). Defects in cross-regulation, or in the formation or maintenance of cerebellar germinal zones may result in cerebellar hypoplasia by directly disrupting the formation of cerebellar neurons, or by undermining subsequent interactions that lead to the massive expansion of the granule cell precursor pool in the EGL.

#### Progenitor cell migration, proliferation, and differentiation

Tissue growth in the developing embryo has to be tightly regulated to allow the coordinated expansion of different cell types. Coordinated growth requires communication between two or more closely apposed tissue or cell layers. Perhaps the best-known example is the orchestration of epithelial growth and morphogenesis through epithelial–mesenchymal interactions. Postnatal cerebellar growth is regulated in a similar manner. Rapid cerebellar growth is primarily driven by the proliferation of GCps in the EGL, a process largely coordinated by a layer of Purkinje neurons under the surface of the cerebellum (Hatten and Heintz, 1995). As we have discussed, the failure to specify Purkinje neurons is associated with severe cerebellar hypoplasia. After their birth in the VZ, Purkinje neuron progenitors migrate along radial glia toward the pial surface of the cerebellar anlage. Genetic defects that disrupt the glial scaffold, or the production of signals and cell-intrinsic mechanisms that control Purkinje cell migration result in various degrees of cerebellar hypoplasia (**Figure 2B**). In addition, cell migration defects resulting in the ectopic localization of Purkinje cells are likely to underlie many examples of cerebellar heterotopias (Yang et al., 2002).

One of the central pathways linked to GCp proliferation and differentiation is the SHH pathway. Immature Purkinje cells secrete SHH and that the proliferation of GCps is critically dependent on SHH signaling (Dahmane and Ruiz i Altaba, 1999; Wallace, 1999; Wechsler-Reya and Scott, 1999). In mouse, conditional deletion of *Shh* from PCs (Purkinje cells) or SHH signal transduction components like *Smo* (smoothened), *Gli1*, and *Gli2* from GCps have all been shown to result in defects in GCp proliferation and cerebellar hypoplasia (Lewis et al., 2004; Corrales et al., 2006; Spassky et al., 2008). Disrupting PC migration or differentiation result in similar phenotypes (Hatten, 1999). For example, mice homozygous for the reeler allele, *Reln*^rl/rl^, exhibit severe cerebellar hypoplasia (Magdaleno et al., 2002). In the absence of Reelin, PCs fail to organize in the form of a Purkinje plate under the pial surface of the E14.5 cerebellum (Mariani et al., 1977; Miyata et al., 1997). These observations suggest that the primary cause of cerebellar hypoplasia associated with defects in Reelin signaling is the failure of SHH-expressing PCs to reach their appropriate position underneath the EGL where they provide a proliferative SHH signal.

It is important to note that cerebellar hypoplasia caused by defects in SHH signaling affects the (primarily postnatal) proliferation of GCps in the vermis and hemispheres equally, resulting in a phenotype that differs significantly from early IsO defects with disproportionally hypoplastic vermis.

Conditional manipulation of signaling pathways that function during early cerebellar development have revealed additional functions during later stages of cerebellar development. Again, the WNT and FGF pathways provide good examples of this principle. Reduced WNT signaling during early development result in cerebellar defects typical of reduced IsO function, i.e., vermis aplasia (see The Isthmus Organizer). WNT/β-catenin signaling is also active at later stages of cerebellar development, particularly in the germinal zones and Bergmann glia (Selvadurai and Mason, 2011). The role of WNT/β-catenin signaling at later developmental stages has been investigated more recently. Several groups have reported that increased β-catenin signaling can alter the proliferation and differentiation of neuronal progenitors in the developing cerebellum. Lorenz et al. (2011) showed that activation of WNT/β-catenin signaling by deletion of the *Apc* (adenomatous polyposis coli) gene or stabilization of β-catenin in GCps inhibited their proliferation and enhanced their differentiation. As a consequence, these mice exhibited cerebellar hypoplasia and ataxia. The Wechsler-Reya group confirmed the potent inhibition of GCp proliferation and cerebellar hypoplasia as a result of hyperactivation of β-catenin in GCps, but found additionally that the proliferation of GABA-ergic progenitors in the VZ was significantly increased when signaling was increased in this progenitor zone (Pei et al., 2012). The observation of differential effects of increased WNT signaling on progenitors in different anatomical locations is also important in understanding the origins of childhood tumors of the cerebellum, medulloblastoma. Medulloblastoma subtypes characterized by activating mutations in WNT pathway genes are most likely to originate from the lower, i.e., non-cerebellar RL and not from cells in the EGL (Gibson et al., 2010), in agreement with the finding that WNT activation inhibits cell proliferation in the EGL.

Similar complexities have been observed upon altering FGF signaling during cerebellar development. Increased FGF signaling upon deletion of Sprouty genes, which encode FGF antagonists, have opposite effects on cerebellar growth depending on the time of deletion. Upregulated FGF signaling during early development is associated with the expansion of vermis progenitors in anterior r1 and an expansion of the width of the vermis. Increased FGF signaling during late embryonic and postnatal stages on the other hand is associated with abnormalities in Bergmann glial and Purkinje cell differentiation, reduced SHH production by these cells as well as an inhibitory effect on the responsiveness of GCps to SHH. As a consequence of these changes, GCps prematurely exit the cell cycle and differentiate, leading to general cerebellar hypoplasia (Yu et al., 2011).

These observations provide a window on some of the complexities of understanding the role of particular pathways in cerebellar development. Not only does one pathway have multiple functions at different developmental time points, the effects of deregulated signaling in one neuronal progenitor subpopulation can be vastly different from another. In the light of these observations, any attempt to infer mechanistic explanations for cerebellar phenotypes from observations made in unrelated cell types or simplified *in vitro* cultures have to be interpreted with considerable caution. Although these studies are useful as initial proof-of-principle studies, validation and analysis in the appropriate cell types in an intact developing cerebellum are essential.

### MECHANISTIC INSIGHTS INTO THE CAUSES OF HUMAN CEREBELLAR DEFECTS

Insights gained from studies in model organisms have greatly improved our ability to identify the underlying processes responsible for many congenital cerebellar anomalies that affect the human population. In this section, we aim to demonstrate some of these new insights by discussing our current understanding of two major syndromes associated with cerebellar hypoplasia: Joubert syndrome and Dandy–Walker malformation. We contrast the remarkable progress that has been made in our understanding of these syndromes with pontocerebellar hypoplasia, a group of conditions for which candidate genes have been identified but little mechanistic insights are available.

#### Disrupted WNT signaling in joubert syndrome

Joubert syndrome is characterized by cerebellar vermis hypoplasia and abnormal superior cerebellar peduncles and associated with both movement disorders and mental retardation (Joubert et al., 1999). Joubert syndrome has been the subject of intense study by human geneticists and more than 20 disease-associated genes have been identified to date (Valente et al., 2013). All these genes appear to encode proteins that are associated with the assembly and biology of cilia, and Joubert syndrome can therefore be classified within the larger group of ciliopathies, alongside Bardet–Biedl and Meckel syndromes (Brancati et al., 2010).

Despite the wealth of human genetics data and direct links to ciliary assembly and function, the cilia-dependent developmental pathways disrupted in Joubert syndrome are poorly understood. A recent study by the Gleeson group provided important insights into the causes of cerebellar phenotypes in Joubert syndrome caused by mutations in *AHI1* (Abelson helper integration site 1). First, they showed convincingly that vermis hypoplasia in *Ahi1-/-* mouse mutants is associated with an expanded roof plate and a vermis midline “fusion” defect (Lancaster et al., 2011). This cerebellar midline defect phenocopies the vermis agenesis characteristic of mutants with reduced IsO function like *Wnt1*^sw/sw^ and FGF hypomorphs (Louvi et al., 2003; Basson et al., 2008). From studies in the mouse, we would hypothesize that the apparent “fusion” defect in these mutants is primarily caused by the failure of vermis progenitors in the anterior cerebellar anlage to expand, a defect that is likely associated with the abnormal expansion of roof plate tissue (Basson et al., 2008). WNT signaling was not measured in *Ahi1-/-* embryos at early stages when the IsO is functional (E9.5–E12.5) and roof plate expansion at the expense of the expansion of vermis progenitors has been shown to take place in FGF pathway mutants. However, WNT signaling was diminished in the medial cerebellar anlage at E13.5, which correlated with reduced proliferation of vermis progenitors adjacent to the midline. Finally, WNT/β-catenin activation by LiCl treatment at E12.5 and E13.5 partially rescued the proliferation defect and midline “fusion,” confirming reduced WNT signaling as an important pathogenic mechanism in Joubert syndrome (Lancaster et al., 2009). A similar, yet milder phenotype is also reported in *Cep290-/-* animals. Although WNT signaling was not investigated in the *Cep290* (centrosomal protein 290 kDa) mutants, the data suggest that a WNT signaling defects might also underlie Joubert syndrome caused by mutations in Joubert-associated genes other than *AHI1*. This study clearly implicates deregulated WNT signaling as a mechanism in Joubert syndrome.

Abdelhamed et al. (2013) recently reported some interesting observations implicating the Joubert syndrome gene, *Tmem67* (transmembrane protein 67) in the regulation of SHH and WNT signaling. The authors report a significant expansion of the fourth ventricle roof plate in *Tmem67-/-* embryos, a phenotype consistent with a disruption of early IsO function and vermis hypoplasia/aplasia. Based on the studies discussed so far, one might predict that reduced WNT signaling in these mutants may lead directly to reduced activity of the IsO, early loss of vermis progenitors, or reduced proliferation of cells at the midline, akin to the hypothesis to explain vermis defects in *Ahi-/-* embryos. Indeed, the authors present data to suggest that WNT/β-catenin signaling is reduced in *Tmem67-/-* embryonic fibroblasts from embryos with Joubert syndrome-like phenotypes. They also report reduced *Shh* expression in the ventral neural tube. It will be important to examine WNT and SHH signaling in mes/r1 in *Tmem67* mutants, in order to draw direct conclusions as to the involvement of these pathways in cerebellar development at these critical early developmental stages. Taken together, these recent studies suggest that several signaling pathways might be affected as a result of defective ciliary function and that the cerebellar vermis phenotype in Joubert syndrome is due to the deregulation of signaling pathways required for early IsO function and vermis expansion.

In addition to the well-known WNT and SHH pathways, the non-canonical WNT-planar cell polarity (PCP) pathway has also been implicated in the pathogenesis of Joubert syndrome. Knockdown or mutation of *Tmem216* (transmembrane protein 216), a gene mutated in Joubert and Meckel syndromes, resulted in ciliogenesis defects, and impaired centrosome docking to cilia due to the mislocalization of hyperactivated RhoA. Disheveled1 (Dvl1) phosphorylation in response to *Tmem216* knockdown and mild PCP phenotypes were observed in zebrafish (Valente et al., 2010). The role of WNT-PCP pathway during cerebellar morphogenesis has not been investigated and the developmental processes that might be sensitive to disrupted WNT-PCP signaling remains an important question to address in cerebellar development.

Although the critical roles of FGF signaling during cerebellar development are well-established in model organisms, FGF signaling defects have not been linked directly to cerebellar vermis defects in humans. This might be due to the fact that FGF hypomorphic mutations that reduce FGF signaling sufficiently to cause a cerebellar vermis phenotype, are also detrimental to the development of many other organs, such that foetuses carrying these mutations will not survive past birth. Two groups have reported rare patients with Kallmann syndrome and partial cerebellar vermis a/hypoplasia, initially diagnosed as Dandy–Walker malformation (Ueno et al., 2004; Aluclu et al., 2007). Mutations in the coding sequence of *KAL1* (Kallmann syndrome 1 sequence) was excluded by Ueno et al. in their study, leaving the possibility that mutations in other Kallmann syndrome-associated genes that affect FGF signaling might be responsible.

#### Dandy–Walker malformation

Dandy–Walker malformation of the cerebellum is diagnosed upon the identification of vermis hypoplasia (Brodal, 1945), rotation of the vermis away from the brain stem and an enlarged posterior fossa. Behavioral pathology can include motor deficits consistent with cerebellum damage and, in 50% of patients, intellectual impairment that has been tentatively correlated with the degree of loss of vermal lobulation (Boddaert et al., 2003). Grinberg et al. (2004) identified the linked *ZIC1* (zinc finger protein of the cerebellum 1) and *ZIC4* (zinc finger protein of the cerebellum 4) genes on 3q24 as candidate genes, an observation since confirmed by other groups (Tohyama et al., 2011). Experiments in *Zic1-/-;Zic4-/-* mouse models indicated that these genes were required for the full responsiveness of GCps to SHH. *Zic1-/-;Zic4-/-* GCps correspondingly showed reduced proliferation and general cerebellar hypoplasia. These cerebella also showed the loss of the anterior folium in the vermis that was specifically due to the loss of *Zic1*, which is uniquely expressed in the VZ. Taken together, this study links *Zic1* and *Zic4* to the SHH pathway and GCps proliferation postnatally, and suggests that additional genetic or perhaps non-genetic factors are responsible for causing the pronounced vermis a/hypoplasia characteristic of Dandy–Walker malformation, perhaps by interacting with *Zic1* (Blank et al., 2011).

The analysis of *Foxc1* (forkhead box C1) mouse mutants after the identification of *FOXC1* as a candidate gene for Dandy–Walker malformation in humans, has revealed an novel mechanism whereby cerebellar vermis hypoplasia could arise. Mouse*Foxc1* hypomorphs exhibit vermis hypoplasia confirming that reduced FOXC1 function is responsible for Dandy–Walker malformation in humans. Intriguingly, *Foxc1* is not expressed in the developing cerebellum, but in mesenchymal tissue of the posterior fossa covering the cerebellar anlage from about E11.5. The IsO, roof plate, and RL initially develop normally in these mutants, but *Atoh1* expression in GCps in the medial EGL was lost by E14.5 of development. As *Atoh1* is required for GCp proliferation (Flora et al., 2009), these cells fail to expand resulting in the absence of an EGL in the medial cerebellum by birth. The exact mechanisms whereby FOXC1 in the cranial mesenchyme controls *Atoh1* expression in GCps are not known, but Aldinger et al. (2009) showed that the expression of BMP and TGFβ family genes are reduced in these mutant embryos. As BMP signaling is required for normal *Atoh1* expression and GCp expansion, this observation provides a likely explanation for vermis hypoplasia in patients with *FOXC1* mutations (Tong and Kwan, 2013). These findings highlight the important contribution of signaling interactions between progenitor zones and non-progenitor tissues like the cranial mesenchyme and roof plate in cerebellar development.

In conclusion, studies so far appear to primarily link Dandy–Walker Malformations to defects in GCp expansion. However, with the exception of *Foxc1*, the reasons for the disproportionate effect on vermis progenitors are not understood. It is interesting to note that both *Zic1* and *Zic4* are also expressed in the mesenchyme overlying the cerebellar anlage; perhaps these genes have an additional function in this tissue that might explain the vermis hypoplasia (Blank et al., 2011).

#### Pontocerebellar hypoplasia

Pontocerebellar hypoplasia is characterized by hypoplasia of the brainstem and cerebellum by birth; a condition to usually deteriorates further suggesting that some pontocerebellar hypoplasias can be classified as a neurodegenerative condition. Clinical signs include severe motor and developmental delays, respiratory deficiency and early postnatal lethality (Barth, 1993). The *CHMP1A* (charged multivesicular body protein 1a) gene that encodes chromatin modifying protein 1A (CHMP1A), has been identified as a candidate gene for pontocerebellar hypoplasia. Patient-derived lymphoblastoid cell lines showed reduced proliferation and increased expression of the cell cycle inhibitor INK4A (cyclin-dependent kinase inhibitor 2A, p16Ink4a), a target of the Polycomb group member BMI1 (B lymphoma Mo-MLV insertion region 1 homolog). This change in expression correlated with reduced BMI1 recruitment to an *Ink4a* regulatory region, suggesting that CHMP1A may regulate Polycomb recruitment to gene loci, thereby phenocopying the cerebellar phenotype in *Bmi1-/-* mutants and (Mochida et al., 2012). To what extent the loss of BMI1 or other Polycomb components affects the development of pontine structures have not been established.

With the rapid advance in genomic science many genetic changes have now been identified to be associated with human pontocerebellar hypoplasia. These include intriguing candidates like *CASK* (calcium/calmodulin-dependent serine protein kinase)*, EXOSC3* (exosome component 3)*, RARS2* (arginyl-tRNA synthetase 2), *TSEN54* (tRNA-splicing endonuclease subunit 54), *TSEN2* (tRNA-splicing endonuclease subunit 2), and *TSEN34 *(tRNA-splicing endonuclease subunit 34; Edvardson et al., 2007; Najm et al., 2008; Graham et al., 2010; Wan et al., 2012). The latter genetic associations suggest pathogenic mechanism related to defects in fundamental processes like protein synthesis and RNA splicing, but the mechanisms are likely to be varied and largely remain unexplored.

## COGNITIVE CONSEQUENCES OF CEREBELLAR DEFECTS

In addition to well-established effects on motor coordination, the consequences of cerebellar defects on non-motor behaviors have received increasing attention over the last 20 years (Strick et al., 2009). Cerebellar lesions like those acquired after tumor resection have been linked to a number of non-motor behavioral disturbances. Schmahmann and Sherman (1998) coined the term “cerebellar cognitive affective syndrome” to describe the range of behavioral alterations associated with cerebellar lesions. These include alterations in (i) executive functions like planning and abstract reasoning, (ii) spatial recognition, (iii) language difficulties especially with grammar and controlling pitch and timing, and (iv) changes in measures of personality like emotions and social behavior (Schmahmann and Sherman, 1998; Tavano et al., 2007). Attempts to conceptualize this constellation of phenotypes have focused on the capacity of the cerebellum to accurately measure time intervals (Ivry et al., 1988; Ackermann et al., 1998; Schmahmann, 1998). The demonstrable ability of the cerebellum to generate internal models during learning (Imamizu et al., 2000) might thus be applied to generating internal models of cognitive processes via a series of re-entrant connections with non-motor cortical areas (Schmahmann and Pandya, 1991).

The anatomical basis for the influence of the cerebellum on cortical activity is the ascending projection from the dentate nucleus: a deep cerebellar nuclear formation that appears to be exclusive to mammals (Nieuwenhuys et al., 1998). The output of the dentate nucleus is modulated by Purkinje cells in the large neo-cerebellar hemispheres. In these regions the relatively coarse correspondence of cerebellar topography with particular body regions breaks down and is, at best, highly fragmented (Manni and Petrosini, 2004). In recent years, the advent of new trans-synaptic labeling techniques has allowed these cortical connections in primates, which were identified by traditional neuronal tracers (Schmahmann and Pandya, 1989), to be mapped with greatly improved precision. This has revealed that both primary motor cortex and dorsolateral prefrontal cortex are connected in “closed” loops with the cerebellum. In other words, Purkinje cells that modulate these cortical areas via the dentate nucleus, receive a reciprocal cortical input from the same regions via the pons (Kelly and Strick, 2003; **Figure 2C**). Furthermore, and contrary to prevailing theories (Glickstein, 2000), there is little anatomical cross-talk between these distinct loops. These observations sketch out a system of cerebello-cortical connectivity where substantial areas of the cerebellum are anatomically allocated to distinct cognitive processes. It is perhaps therefore unsurprising that the ventral portion of the dentate nucleus, which has been proposed to specifically mediate “cognitive” connections (Strick et al., 2009) is substantially greater in humans than other primates (Matano, 2001).

## THE CEREBELLUM AND AUTISTIC SPECTRUM DISORDER

The increasing evidence for a cognitive role for the cerebellum has correlated with a number of studies that have linked forms of cerebellar hypoplasia with ASD (Courchesne et al., 1988, 1994; Bauman, 1991; Courchesne, 1997; Palmen et al., 2004; Dicicco-Bloom et al., 2006; Schmahmann, 2010; Fatemi et al., 2012). While firmly located within cortical processes (Mundy, 2003), these studies have prompted the hypothesis that ASD, might be mediated in part by interactions between cerebellum and cortex. By analogy, ASD represents a deficit in the cognitive equivalent of the modulatory processes by which the cerebellum has long been know to fine-tune motor skills and learning: a so-called “dysmetria of thought” (Schmahmann, 2010).

While this area of theory is necessarily in its early stages of development, it defines a new frontier for cerebellar research that has been probed both in human studies and experimentally in mice. Various groups have identified cerebellar vermis hypoplasia in patients with syndromic forms of autism (Becker et al., 2001; Sanlaville and Verloes, 2007; Aldinger et al., 2013). However, the precise link between cerebellar development and ASD are unclear. Studies of large-scale cerebellar pathology have revealed conflicting data with respect to the tempo of brain growth in patients and control groups (reviewed by Palmen et al., 2004). By contrast, at a cellular level, more substantive trends have emerged in terms of Purkinje cell loss (Ritvo et al., 1986; Kemper and Bauman, 1993) or reduction in cell size (Fatemi et al., 2002). Furthermore, some studies have shown an intriguing specificity in the location of Purkinje cell loss (Courchesne et al., 1988) with respect to areas activated during cognitive processes (Stoodley and Schmahmann, 2009). A small number of studies also examined specific alterations in cerebellar nucleus connections as assessed by diffusion tensor imaging (Brito et al., 2009; Sivaswamy et al., 2010) and histopathology (Bauman and Kemper, 1994). In particular, the superior (containing cerebellar output to thalamus) and middle (input from pons) cerebellar peduncles, which underlie cortical cerebellar loops, appear specifically affected.

From these findings it is apparent that ASD is accompanied by pathology in different cell types reflected by different developmental origins within different time windows. This fragmented pattern of pathology might reflect multiple different developmental aetiologies for a syndrome that is represented by a broad range of severity of disability. Alternatively, these observations may reflect coordinated patterns of trans-synaptic degeneration stemming from a single locus. The flipside to this perspective is retrograde neurodegeneration might predict patterns of cell loss that help establish the timing for developmental causes of ASD. As Bauman and Kemper (2005) point out, the lack of retrograde loss of olivary neurons in ASD patients with reduced numbers of Purkinje cells suggests that cell death must have occurred prior to 28–30 weeks gestation (Bauman and Kemper, 2005).

Despite the potential complexities of cerebellar origins of ASD, a recent analysis of an ASD-like condition in a tuberous sclerosis complex 1 (*Tsc1*) mutant mouse is a particularly significant step in linking cerebellar defects to ASD (Tsai et al., 2012). Firstly, it establishes the parameters of a reference mouse model for human ASD, which is exhibited by many Tuberous Sclerosis patients. Secondly, Sahin and colleagues generated a mutant where gene deletion was restricted to the cerebellum and yet still recapitulated ASD-like behavior. This provides compelling evidence that developmental defects restricted to the cerebellum can result in a number of behaviors typical of autism. By deleting *Tsc1* only from cerebellar Purkinje cells during postnatal development, Tsai et al. (2012) could show that a reduction in Purkinje cells and increased Purkinje cell spine density were associated with altered behaviors typically associated with ASDs. The core behavioral features assessed during ASD diagnosis in humans include deficits in social reciprocity, communication, language delay, repetitive behaviors, and an insistence on sameness. Some of these behaviors can be modeled and was examined in these Purkinje cell-specific *Tsc1* mutant mice. These mice scored low on sociability and social novelty tests when presented with the opportunity to interact with a new mouse. Although communication and language are not easily assessed in mice, pups will attempt to attract the mother’s attention by ultrasonic vocalizations when separated from her. *Tsc1* mutant pups showed an increase in these vocalizations, suggesting that the lack of *Tsc1* in the cerebellum can affect this process, although the relevance of this observation to ASDs is not clear. Finally, *Tsc1*-deficient mice exhibited excessive grooming, an indication of repetitive behaviors and demonstrated cognitive inflexibility in a reversal learning paradigm, perhaps indicative of an insistence of sameness (Tsai et al., 2012). This landmark study provides direct evidence that subtle disruptions in cerebellar architecture can have pronounced effects on behaviors typically associated with cortical defects. The promise of such mouse models lies not only in understanding the genetic basis of ASD but also the anatomical questions of where in the anatomical pathways of cortico-cerebellar connectivity gene deletions impose circuit-wide pathology.

## FUTURE DIRECTIONS

The identification of the genetic defects responsible for specific anatomical abnormalities in the cerebellum is likely to continue at an unrivalled speed owing largely to the revolution in next generation sequencing. However, it should be remembered that knowledge of the genetic basis of disease is only the first step in understanding the condition. The next big challenge is to unravel the developmental and molecular mechanisms by which genetic changes manifest in a particular disease phenotype. Studies in model systems like the mouse have been essential in this quest and will no doubt continue to remain so. Without the fundamental knowledge gained from these experimental studies, translation of genetic findings to preventative and curative strategies will not be possible.

Unraveling the salient features and functional importance of cerebellar connectivity with cortical regions implicated in psychiatric conditions like autism, ADHD and schizophrenia, is of the utmost importance of we want to understand the involvement of the cerebellum in these conditions (Whitty et al., 2009; O’Halloran et al., 2012). Multi-disciplinary teams that combine the expertise of clinicians, radiologists, human geneticists, developmental and molecular biologists, and experts in rodent behavior are most likely to succeed in providing a more complete understanding of how genes and mechanisms that control cerebellar development relate to cerebellar disease and function.

Significant differences between the neuroanatomy of rodents and primates may emerge as barriers to understanding cognitive deficits in the mouse, despite all its advantages as an experimental and genetic model. Nevertheless, we highlight here that recent insights gained in the Tsc1 mutant suggest that the substrates for cortico-cerebellar modulation of behavior are present in the rodent. While this field is relatively undeveloped, the promise for understanding the full range of cerebellum function through exploration of these systems in mouse presents a research avenue of enormous promise.

Finally, this article almost exclusively focuses on cerebellar hypoplasia. Many other congenital cerebellar malformations have been described, including a range of conditions that appear to be associated with malformation of the posterior fossa and cerebellar overgrowth. For example, rasopathies like Costello syndrome are characterized by Chiari type I malformation and cerebellar tonsillar herniation (Gripp et al., 2010). One might like to speculate that abnormal signaling between the cranial mesenchyme and the cerebellar anlage, as those implicated in the aetiology of Dandy–Walker malformation could be involved in a subset of these conditions. However, the underlying mechanisms responsible for cerebellar defects in these conditions remain largely enigmatic. Epigenetic and environmental influences are also likely to contribute substantially to cerebellar disease burden and psychiatric diseases linked to cerebellar dysfunction. Investigative studies in the laboratory, aimed at unraveling the potential mechanisms whereby alterations that affect development through means other than changes in the coding sequences of genes cause disease are just beginning and represent an important piece of the puzzle (Pidsley et al., 2012; James et al., 2013).

## Conflict of Interest Statement

The authors declare that the research was conducted in the absence of any commercial or financial relationships that could be construed as a potential conflict of interest.

## Abbreviations

Ahi1: Abelson helper integration site 1
Apc: adenomatous polyposis coli
Atoh1: atonal homolog 1
BMI1: B lymphoma Mo-MLV insertion region 1 homolog
CASK: calcium/calmodulin-dependent serine protein kinase
Cep290: centrosomal protein 290 kDa
CHMP1A: charged multivesicular body protein 1a
EGL: external granule cell (germinal) layer
En1: engrailed 1
En2: engrailed 2
EXOSC3: exosome component 3
FGF: fibroblast growth factor
Foxc1: forkhead box C1
Gbx2: gastrulation brain homeobox 2
GCps: granule cell precursors
Gli: GLI-Kruppel family member
Ink4a: cyclin-dependent kinase inhibitor 2A, p16Ink4a
IsO: isthmus organizer
KAL1: Kallmann syndrome 1 sequence
LMX1B: Lim homeobox transcription factor 1 beta
Otx2: orthodenticle homeobox 2
Pax2: paired box gene 2
PC: Purkinje cell
Ptf1a: pancreas transcription factor 1 subunit alpha
r1: rhombomere 1
RARS2: arginyl-tRNA synthetase 2
Reln: Reelin
RL: rhombic lip
Shh: Sonic Hedgehog
Smo: smoothened
SUFU: suppressor of fused homolog
TGFβ: transforming growth factor beta
Tmem67: transmembrane protein 67
Tmem216: transmembrane protein 216
Tsc1: tuberous sclerosis complex 1
TSEN2: tRNA-splicing endonuclease subunit 2
TSEN34: tRNA-splicing endonuclease subunit 34
TSEN54: tRNA-splicing endonuclease subunit 54
vz: ventricular zone
Wnt1: wingless-type MMTV integration site family, member 1
ZIC1: zinc finger protein of the cerebellum 1
ZIC3: zinc finger protein of the cerebellum 3
ZIC4: zinc finger protein of the cerebellum 4.

## References

[B1] Abdelhamed Z. A. Wheway G. Szymanska K. Natarajan S. Toomes C. Inglehearn C. (2013). Variable expressivity of ciliopathy neurological phenotypes that encompass Meckel–Gruber syndrome and Joubert syndrome is caused by complex de-regulated ciliogenesis, Shh and Wnt signalling defects. *Hum. Mol. Genet.* 22 1358–1372 10.1093/hmg/dds546 23283079 PMC3596847

[B2] Ackermann H. Wildgruber D. Daum I. Grodd W. (1998). Does the cerebellum contribute to cognitive aspects of speech production? A functional magnetic resonance imaging (fMRI) study in humans. *Neurosci. Lett.* 247 187–190 10.1016/S0304-3940(98)00328-0 9655624

[B3] Alder J. Cho N. K. Hatten M. E. (1996). Embryonic precursor cells from the rhombic lip are specified to a cerebellar granule neuron identity. *Neuron* 17 389–399 10.1016/S0896-6273(00)80172-5 8816703

[B4] Aldinger K. A. Kogan J. Kimonis V. Fernandez B. Horn D. Klopocki E. (2013). Cerebellar and posterior fossa malformations in patients with autism-associated chromosome 22q13 terminal deletion. *Am. J. Med. Genet. A* 161A 131–136 10.1002/ajmg.a.35700 23225497 PMC3733662

[B5] Aldinger K. A. Lehmann O. J. Hudgins L. Chizhikov V. V. Bassuk A. G. Ades L. C. (2009). FOXC1 is required for normal cerebellar development and is a major contributor to chromosome 6p25.3 Dandy–Walker malformation. *Nat. Genet. * 41 1037–1042 10.1038/ng.422 19668217 PMC2843139

[B6] Aluclu M. U. Bahceci S. Bahceci M. (2007). A rare embryological malformation of brain – Dandy–Walker syndrome – and its association with Kallmann’s syndrome. *Neuro Endocrinol. Lett.* 28 255–258 17627258

[B7] Aoto K. Nishimura T. Eto K. Motoyama J. (2002). Mouse GLI3 regulates Fgf8 expression and apoptosis in the developing neural tube, face, and limb bud. *Dev. Biol.* 251 320–332 10.1006/dbio.2002.0811 12435361

[B8] Baala L. Romano S. Khaddour R. Saunier S. Smith U. M. Audollent S. (2007). The Meckel–Gruber syndrome gene, MKS3, is mutated in Joubert syndrome. *Am. J. Hum. Genet.* 80 186–194 10.1086/510499 17160906 PMC1785313

[B9] Barkovich A. J. Millen K. J. Dobyns W. B. (2009). A developmental and genetic classification for midbrain–hindbrain malformations. *Brain* 132 3199–3230 10.1093/brain/awp247 19933510 PMC2792369

[B10] Barlow J. (2002). *The Cerebellum and Adaptive Control*. Cambridge: Cambridge University Press

[B11] Barth P. G. (1993). Pontocerebellar hypoplasias. An overview of a group of inherited neurodegenerative disorders with fetal onset. * Brain Dev.* 15 411–422 10.1016/0387-7604(93)90080-R 8147499

[B12] Basson M. A. Echevarria D. Ahn C. P. Sudarov A. Joyner A. L. Mason I. J. (2008). Specific regions within the embryonic midbrain and cerebellum require different levels of FGF signaling during development. *Development* 135 889–898 10.1242/dev.011569 18216176 PMC2555978

[B13] Bauman M. L. (1991). Microscopic neuroanatomic abnormalities in autism. *Pediatrics* 87 791–796 2020538

[B14] Bauman M. L. Kemper T. L. (1994). “Neuroanatomic observations of the brain in autism,” in *The Neurobiology of Autism* eds Bauman M. L. Kemper T. L. (Baltimore: Johns Hopkins University Press) 119–145

[B15] Bauman M. L. Kemper T. L. (2005). Neuroanatomic observations of the brain in autism: a review and future directions. *Int. J. Dev. Neurosci.* 23 183–187 10.1016/j.ijdevneu.2004.09.006 15749244

[B16] Becker R. Stiemer B. Neumann L. Entezami M. (2001). Mild ventriculomegaly, mild cerebellar hypoplasia and dysplastic choroid plexus as early prenatal signs of CHARGE association. *Fetal Diagn. Ther.* 16 280–283 10.1159/000053928 11509849

[B17] Ben-Arie N. Bellen H. J. Armstrong D. L. Mccall A. E. Gordadze P. R. Guo Q. (1997). Math1 is essential for genesis of cerebellar granule neurons. *Nature* 390 169–172 10.1038/36579 9367153

[B18] Blaess S. Stephen D. Joyner A. L. (2008). Gli3 coordinates three-dimensional patterning and growth of the tectum and cerebellum by integrating Shh and Fgf8 signaling. *Development* 135 2093–2103 10.1242/dev.015990 18480159 PMC2673693

[B19] Blank M. C. Grinberg I. Aryee E. Laliberte C. Chizhikov V. V. Henkelman R. M. (2011). Multiple developmental programs are altered by loss of Zic1 and Zic4 to cause Dandy–Walker malformation cerebellar pathogenesis. *Development* 138 1207–1216 10.1242/dev.054114 21307096 PMC3042874

[B20] Boddaert N. Klein O. Ferguson N. Sonigo P. Parisot D. Hertz-Pannier L. (2003). Intellectual prognosis of the Dandy–Walker malformation in children: the importance of vermian lobulation. *Neuroradiology* 45 320–324 10.1007/s00234-003-0980-6 12682795

[B21] Bottmer C. Bachmann S. Pantel J. Essig M. Amann M. Schad L. R. (2005). Reduced cerebellar volume and neurological soft signs in first-episode schizophrenia. *Psychiatry Res.* 140 239–250 16288852 10.1016/j.pscychresns.2005.02.011

[B22] Braitenberg V. Atwood R. P. (1958). Morphological observations on the cerebellar cortex. *J. Comp. Neurol.* 109 1–34 10.1002/cne.901090102 13563670

[B23] Brancati F. Dallapiccola B. Valente E. M. (2010). Joubert Syndrome and related disorders. *Orphanet J. Rare Dis.* 5 20 10.1186/1750-1172-5-20 PMC2913941 20615230

[B24] Brault V. Moore R. Kutsch S. Ishibashi M. Rowitch D. H. Mcmahon A. P. (2001). Inactivation of the beta-catenin gene by Wnt1-Cre-mediated deletion results in dramatic brain malformation and failure of craniofacial development. *Development* 128 1253–1264 11262227 10.1242/dev.128.8.1253

[B25] Brito A. R. Vasconcelos M. M. Domingues R. C. Hygino Da Cruz L. C. Jr., Rodrigues Lde S. Gasparetto E. L. (2009). Diffusion tensor imaging findings in school-aged autistic children. *J. Neuroimaging* 19 337–343 10.1111/j.1552-6569.2009.00366.x 19490374

[B26] Broccoli V. Boncinelli E. Wurst W. (1999). The caudal limit of Otx2 expression positions the isthmic organizer. *Nature* 401 164–168 10.1038/43670 10490025

[B27] Brodal A. (1945). Defective development of the cerebellar vermis (partial agenesis) in a child. *Skr. Norske Vidensk.-Akad. I. Mat.-Nat.* K1.3 1–40

[B28] Broom E. R. Gilthorpe J. D. Butts T. Campo-Paysaa F. Wingate R. J. (2012). The roof plate boundary is a bi-directional organiser of dorsal neural tube and choroid plexus development. *Development* 139 4261–4270 10.1242/dev.082255 23052907 PMC3478690

[B29] Cajal S. R. Y. (1894). *Les Nouvelles Idéees sur la Structure du Système Nerveux chez l’Homme et chez les Vertébrés.* Paris: C. Reinwald & Cie 10.5962/bhl.title.48561

[B30] Cheng Y. Sudarov A. Szulc K. U. Sgaier S. K. Stephen D. Turnbull D. H. (2010). The Engrailed homeobox genes determine the different foliation patterns in the vermis and hemispheres of the mammalian cerebellum. *Development* 137 519–529 10.1242/dev.027045 20081196 PMC2858911

[B31] Chi C. L. Martinez S. Wurst W. Martin G. R. (2003). The isthmic organizer signal FGF8 is required for cell survival in the prospective midbrain and cerebellum. *Development* 130 2633–2644 10.1242/dev.00487 12736208

[B32] Chizhikov V. V. Lindgren A. G. Currle D. S. Rose M. F. Monuki E. S. Millen K. J. (2006). The roof plate regulates cerebellar cell-type specification and proliferation. *Development* 133 2793–2804 10.1242/dev.02441 16790481

[B33] Coe B. P. Girirajan S. Eichler E. E. (2012). A genetic model for neurodevelopmental disease. *Curr. Opin. Neurobiol.* 22 829–836 10.1016/j.conb.2012.04.007 22560351 PMC3437230

[B34] Corrales J. D. Blaess S. Mahoney E. M. Joyner A. L. (2006). The level of sonic hedgehog signaling regulates the complexity of cerebellar foliation. *Development* 133 1811–1821 10.1242/dev.02351 16571625

[B35] Corrales J. D. Rocco G. L. Blaess S. Guo Q. Joyner A. L. (2004). Spatial pattern of sonic hedgehog signaling through Gli genes during cerebellum development. *Development* 131 5581–5590 10.1242/dev.01438 15496441

[B36] Courchesne E. (1997). Brainstem, cerebellar and limbic neuroanatomical abnormalities in autism. *Curr. Opin. Neurobiol.* 7 269–278 9142760 10.1016/s0959-4388(97)80016-5

[B37] Courchesne E. Saitoh O. Yeung-Courchesne R. Press G. A. Lincoln A. J. Haas R. H. (1994). Abnormality of cerebellar vermian lobules VI and VII in patients with infantile autism: identification of hypoplastic and hyperplastic subgroups with MR imaging. *AJR Am. J. Roentgenol.* 162 123–130 8273650 10.2214/ajr.162.1.8273650

[B38] Courchesne E. Yeung-Courchesne R. Press G. A. Hesselink J. R. Jernigan T. L. (1988). Hypoplasia of cerebellar vermal lobules VI and VII in autism. *N. Engl. J. Med.* 318 1349–1354 10.1056/NEJM198805263182102 3367935

[B39] Crossley P. H. Martin G. R. (1995). The mouse Fgf8 gene encodes a family of polypeptides and is expressed in regions that direct outgrowth and patterning in the developing embryo. *Development* 121 439–451 7768185 10.1242/dev.121.2.439

[B40] Crossley P. H. Martinez S. Martin G. R. (1996). Midbrain development induced by FGF8 in the chick embryo. *Nature* 380 66–68 10.1038/380066a0 8598907

[B41] Dahmane N Ruiz i Altaba A. (1999). Sonic hedgehog regulates the growth and patterning of the cerebellum. *Development* 126 3089–3100 10375501 10.1242/dev.126.14.3089

[B42] Dicicco-Bloom E. Lord C. Zwaigenbaum L. Courchesne E. Dager S. R. Schmitz C. (2006). The developmental neurobiology of autism spectrum disorder. *J. Neurosci.* 26 6897–6906 10.1523/JNEUROSCI.1712-06.2006 16807320 PMC6673916

[B43] Dixon-Salazar T. Silhavy J. L. Marsh S. E. Louie C. M. Scott L. C. Gururaj A. (2004). Mutations in the AHI1 gene, encoding jouberin, cause Joubert syndrome with cortical polymicrogyria. *Am. J. Hum. Genet.* 75 979–987 10.1086/425985 15467982 PMC1182159

[B44] Eccles J. C. M. Ito M. Szentágothai J. (1967). *The Cerebellum as a Neuronal Machine*. Berlin: Springer-Verlag

[B45] Eddison M. Toole L. Bell E. Wingate R. J. (2004). Segmental identity and cerebellar granule cell induction in rhombomere 1. *BMC Biol.* 2:14 10.1186/1741-7007-2-14 PMC446226 15198802

[B46] Edvardson S. Shaag A. Kolesnikova O. Gomori J. M. Tarassov I. Einbinder T. (2007). Deleterious mutation in the mitochondrial arginyl-transfer RNA synthetase gene is associated with pontocerebellar hypoplasia. *Am. J. Hum. Genet.* 81 857–862 10.1086/521227 17847012 PMC2227936

[B47] Eluvathingal T. J. Behen M. E. Chugani H. T. Janisse J. Bernardi B. Chakraborty P. (2006). Cerebellar lesions in tuberous sclerosis complex: neurobehavioral and neuroimaging correlates. *J. Child Neurol.* 21 846–851 10.1177/08830738060210100301 17005099

[B48] Fatemi S. H. Aldinger K. A. Ashwood P. Bauman M. L. Blaha C. D. Blatt G. J. (2012). Consensus paper: pathological role of the cerebellum in autism. *Cerebellum* 777–807 10.1007/s12311-012-0355-9 22370873 PMC3677555

[B49] Fatemi S. H. Halt A. R. Realmuto G. Earle J. Kist D. A. Thuras P. (2002). Purkinje cell size is reduced in cerebellum of patients with autism. *Cell. Mol. Neurobiol.* 22 171–175 10.1023/A:1019861721160 12363198 PMC11533743

[B50] Ferland R. J. Eyaid W. Collura R. V. Tully L. D. Hill R. S. Al-Nouri D. (2004). Abnormal cerebellar development and axonal decussation due to mutations in AHI1 in Joubert syndrome. *Nat. Genet.* 36 1008–1013 10.1038/ng1419 15322546

[B51] Flora A. Klisch T. J. Schuster G. Zoghbi H. Y. (2009). Deletion of Atoh1 disrupts Sonic Hedgehog signaling in the developing cerebellum and prevents medulloblastoma. *Science* 326 1424–1427 10.1126/science.1181453 19965762 PMC3638077

[B52] Gharani N. Benayed R. Mancuso V. Brzustowicz L. M. Millonig J. H. (2004). Association of the homeobox transcription factor, ENGRAILED 2, 3, with autism spectrum disorder. *Mol. Psychiatry* 9 474–484 10.1038/sj.mp.4001498 15024396

[B53] Gibson P. Tong Y. Robinson G. Thompson M. C. Currle D. S. Eden C. (2010). Subtypes of medulloblastoma have distinct developmental origins. *Nature* 468 1095–1099 10.1038/nature09587 21150899 PMC3059767

[B54] Glickstein M. (2000). How are visual areas of the brain connected to motor areas for the sensory guidance of movement? *Trends Neurosci*. 23 613–617 10.1016/S0166-2236(00)01681-7 11137151

[B55] Graham J. M. Jr., Spencer A. H. Grinberg I. Niesen C. E. Platt L. D. Maya M. (2010). Molecular and neuroimaging findings in pontocerebellar hypoplasia type 2 (PCH2): is prenatal diagnosis possible? *Am. J. Med. Genet. A* 152A 2268–2276 10.1002/ajmg.a.33579 20803644 PMC2931360

[B56] Grinberg I. Northrup H. Ardinger H. Prasad C. Dobyns W. B. Millen K. J. (2004). Heterozygous deletion of the linked genes ZIC1 and ZIC4 is involved in Dandy–Walker malformation. *Nat. Genet.* 36 1053–1055 10.1038/ng1420 15338008

[B57] Gripp K. W. Hopkins E. Doyle D. Dobyns W. B. (2010). High incidence of progressive postnatal cerebellar enlargement in Costello syndrome: brain overgrowth associated with HRAS mutations as the likely cause of structural brain and spinal cord abnormalities. *Am. J. Med. Genet. A* 152A 1161–1168 10.1002/ajmg.a.33391 20425820 PMC4910816

[B58] Guo C. Qiu H. Y. Huang Y. Chen H. Yang R. Q. Chen S. D. (2007). Lmx1b is essential for Fgf8 and Wnt1 expression in the isthmic organizer during tectum and cerebellum development in mice. *Development* 134 317–325 10.1242/dev.02745 17166916

[B59] Hatten M. E. (1999). Central nervous system neuronal migration. *Annu. Rev. Neurosci.* 22 511–539 10.1146/annurev.neuro.22.1.511 10202547

[B60] Hatten M. E. Heintz N. (1995). Mechanisms of neural patterning and specification in the developing cerebellum. *Annu. Rev. Neurosci.* 18 385–408 10.1146/annurev.ne.18.030195.002125 7605067

[B61] Haubold A. Peterson B. S. Bansal R. (2012). Annual research review: progress in using brain morphometry as a clinical tool for diagnosing psychiatric disorders. *J. Child Psychol. Psychiatry* 53 519–535 10.1111/j.1469-7610.2012.02539.x 22394424 PMC4235515

[B62] Holmes G. (1939). The cerebellum of man. *Brain* 62 1–30 10.1093/brain/62.1.1

[B63] Hong S. E. Shugart Y. Y. Huang D. T. Shahwan S. A. Grant P. E. Hourihane J. O. (2000). Autosomal recessive lissencephaly with cerebellar hypoplasia is associated with human RELN mutations. *Nat. Genet.* 26 93–96 10.1038/79246 10973257

[B64] Hoshino M. Nakamura S. Mori K. Kawauchi T. Terao M. Nishimura Y. V. (2005). Ptf1a, a bHLH transcriptional gene, defines GABAergic neuronal fates in cerebellum. *Neuron* 47 201–213 10.1016/j.neuron.2005.06.007 16039563

[B65] Imamizu H. Miyauchi S. Tamada T. Sasaki Y. Takino R. Putz B. (2000). Human cerebellar activity reflecting an acquired internal model of a new tool. *Nature* 403 192–195 10.1038/35003194 10646603

[B66] Ivry R. B. Keele S. W. Diener H. C. (1988). Dissociation of the lateral and medial cerebellum in movement timing and movement execution. *Exp. Brain Res.* 73 167–180 10.1007/BF00279670 3208855

[B67] James S. J. Shpyleva S. Melnyk S. Pavliv O. Pogribny I. P. (2013). Complex epigenetic regulation of Engrailed-2 (EN-2) homeobox gene in the autism cerebellum. *Transl. Psychiatry* 3 e232 10.1038/tp.2013.8 PMC3590998 23423141

[B68] Joubert M. Eisenring J. J. Robb J. P. Andermann F. (1999). Familial agenesis of the cerebellar vermis: a syndrome of episodic hyperpnea, abnormal eye movements, ataxia, and retardation. 1969. *J. Child Neurol.* 14 554–564 10.1177/088307389901400902 10488899

[B69] Joyner A. L. Herrup K. Auerbach B. A. Davis C. A. Rossant J. (1991). Subtle cerebellar phenotype in mice homozygous for a targeted deletion of the En-2 homeobox. *Science* 251 1239–1243 10.1126/science.1672471 1672471

[B70] Joyner A. L. Liu A. Millet S. (2000). Otx2, Gbx2 and Fgf8 interact to position and maintain a mid-hindbrain organizer. *Curr. Opin. Cell Biol.* 12 736–741 10.1016/S0955-0674(00)00161-7 11063941

[B71] Kelly R. M. Strick P. L. (2003). Cerebellar loops with motor cortex and prefrontal cortex of a nonhuman primate. *J. Neurosci.* 23 8432–8444 12968006 10.1523/JNEUROSCI.23-23-08432.2003 PMC6740694

[B72] Kemper T. L. Bauman M. L. (1993). The contribution of neuropathologic studies to the understanding of autism. *Neurol. Clin.* 11 175–187 8441369

[B73] Kiecker C. Lumsden A. (2012). The role of organizers in patterning the nervous system. *Annu. Rev. Neurosci.* 35 347–367 10.1146/annurev-neuro-062111-150543 22462542

[B74] Kim J. J. Gill P. S. Rotin L. Van Eede M. Henkelman R. M. Hui C. C. (2011). Suppressor of fused controls mid-hindbrain patterning and cerebellar morphogenesis via GLI3 repressor. *J. Neurosci.* 31 1825–1836 10.1523/JNEUROSCI.2166-10.2011 21289193 PMC6623745

[B75] Lancaster M. A. Gopal D. J. Kim J. Saleem S. N. Silhavy J. L. Louie C. M. (2011). Defective Wnt-dependent cerebellar midline fusion in a mouse model of Joubert syndrome. *Nat. Med.* 17 726–731 10.1038/nm.2380 21623382 PMC3110639

[B76] Lancaster M. A. Louie C. M. Silhavy J. L. Sintasath L. Decambre M. Nigam S. K. (2009). Impaired Wnt-beta-catenin signaling disrupts adult renal homeostasis and leads to cystic kidney ciliopathy. *Nat. Med.* 15 1046–1054 10.1038/nm.2010 19718039 PMC2895985

[B77] Lewis P. M. Gritli-Linde A. Smeyne R. Kottmann A. Mcmahon A. P. (2004). Sonic hedgehog signaling is required for expansion of granule neuron precursors and patterning of the mouse cerebellum. *Dev. Biol.* 270 393–410 10.1016/j.ydbio.2004.03.007 15183722

[B78] Lo L. Anderson D. J. (2011). A Cre-dependent, anterograde transsynaptic viral tracer for mapping output pathways of genetically marked neurons. *Neuron* 72 938–950 10.1016/j.neuron.2011.12.002 22196330 PMC3275419

[B79] Lorenz A. Deutschmann M. Ahlfeld J. Prix C. Koch A. Smits R. (2011). Severe alterations of cerebellar cortical development after constitutive activation of Wnt signaling in granule neuron precursors. *Mol. Cell. Biol.* 31 3326–3338 10.1128/MCB.05718-11 21690300 PMC3147790

[B80] Louvi A. Alexandre P. Metin C. Wurst W. Wassef M. (2003). The isthmic neuroepithelium is essential for cerebellar midline fusion. *Development* 130 5319–5330 10.1242/dev.00736 14507778

[B81] Machold R. Fishell G. (2005). Math1 is expressed in temporally discrete pools of cerebellar rhombic-lip neural progenitors. *Neuron* 48 17–24 10.1016/j.neuron.2005.08.028 16202705

[B82] Machold R. P. Kittell D. J. Fishell G. J. (2007). Antagonism between Notch and bone morphogenetic protein receptor signaling regulates neurogenesis in the cerebellar rhombic lip. *Neural Dev.* 2 5 10.1186/1749-8104-2-5 PMC1820780 17319963

[B83] Magdaleno S. Keshvara L. Curran T. (2002). Rescue of ataxia and preplate splitting by ectopic expression of Reelin in reeler mice. *Neuron* 33 573–586 10.1016/S0896-6273(02)00582-2 11856531

[B84] Manni E. Petrosini L. (2004). A century of cerebellar somatotopy: a debated representation. *Nat. Rev. Neurosci.* 5 241–249 10.1038/nrn1347 14976523

[B85] Mariani J. (1982). Extent of multiple innervation of Purkinje cells by climbing fibers in the olivocerebellar system of weaver, reeler, and staggerer mutant mice. *J. Neurobiol.* 13 119–126 10.1002/neu.480130204 7062017

[B86] Mariani J. Crepel F. Mikoshiba K. Changeux J. P. Sotelo C. (1977). Anatomical, physiological and biochemical studies of the cerebellum from Reeler mutant mouse. *Philos. Trans. R. Soc. Lond. B Biol. Sci.* 281 1–28 10.1098/rstb.1977.0121 22882

[B87] Martinez S. Crossley P. H. Cobos I. Rubenstein J. L. Martin G. R. (1999). FGF8 induces formation of an ectopic isthmic organizer and isthmocerebellar development via a repressive effect on Otx2 expression. *Development* 126 1189–1200 10021338 10.1242/dev.126.6.1189

[B88] Matano S. (2001). Brief communication: proportions of the ventral half of the cerebellar dentate nucleus in humans and great apes. *Am. J. Phys. Anthropol.* 114 163–165 10.1002/1096-8644(200102)114:2<163::AID-AJPA1016>3.0.CO;2-F 11169906

[B89] McMahon A. P. Bradley A. (1990). The Wnt-1 (int-1) proto-oncogene is required for development of a large region of the mouse brain. *Cell* 62 1073–1085 10.1016/0092-8674(90)90385-R 2205396

[B90] Meek J. (1992). Why run parallel fibers parallel? Teleostean Purkinje cells as possible coincidence detectors, in a timing device subserving spatial coding of temporal differences. *Neuroscience* 48 249–283 10.1016/0306-4522(92)90489-O 1603322

[B91] Meyers E. N. Lewandoski M. Martin G. R. (1998). An Fgf8 mutant allelic series generated by Cre- and Flp-mediated recombination. *Nat. Genet.* 18 136–141 10.1038/ng0298-136 9462741

[B92] Millet S. Bloch-Gallego E. Simeone A. Alvarado-Mallart R. M. (1996). The caudal limit of Otx2 gene expression as a marker of the midbrain/hindbrain boundary: a study using in situ hybridisation and chick/quail homotopic grafts. *Development* 122 3785–3797 9012500 10.1242/dev.122.12.3785

[B93] Millet S. Campbell K. Epstein D. J. Losos K. Harris E. Joyner A. L. (1999). A role for Gbx2 in repression of Otx2 and positioning the mid/hindbrain organizer. *Nature* 401 161–164 10.1038/43664 10490024

[B94] Mishima Y. Lindgren A. G. Chizhikov V. V. Johnson R. L. Millen K. J. (2009). Overlapping function of Lmx1a and Lmx1b in anterior hindbrain roof plate formation and cerebellar growth. *J. Neurosci.* 29 11377–11384 10.1523/JNEUROSCI.0969-09.2009 19741143 PMC2765661

[B95] Miyata T. Nakajima K. Mikoshiba K. Ogawa M. (1997). Regulation of Purkinje cell alignment by reelin as revealed with CR-50 antibody. *J. Neurosci.* 17 3599–3609 9133383 10.1523/JNEUROSCI.17-10-03599.1997 PMC6573700

[B96] Miyata T. Ono Y. Okamoto M. Masaoka M. Sakakibara A. Kawaguchi A. (2010). Migration, early axonogenesis, and Reelin-dependent layer-forming behavior of early/posterior-born Purkinje cells in the developing mouse lateral cerebellum. *Neural Dev.* 5 23 10.1186/1749-8104-5-23 PMC2942860 20809939

[B97] Mochida G. H. Ganesh V. S. De Michelena M. I. Dias H. Atabay K. D. Kathrein K. L. (2012). CHMP1A encodes an essential regulator of BMI1-INK4A in cerebellar development. *Nat. Genet.* 44 1260–1264 10.1038/ng.2425 23023333 PMC3567443

[B98] Mostofsky S. H. Reiss A. L. Lockhart P. Denckla M. B. (1998). Evaluation of cerebellar size in attention-deficit hyperactivity disorder. *J. Child Neurol.* 13 434–439 10.1177/088307389801300904 9733289

[B99] Mundy P. (2003). Annotation: the neural basis of social impairments in autism: the role of the dorsal medial-frontal cortex and anterior cingulate system. *J. Child Psychol. Psychiatry* 44 793–809 10.1111/1469-7610.00165 12959489

[B100] Najm J. Horn D. Wimplinger I. Golden J. A. Chizhikov V. V. Sudi J. (2008). Mutations of CASK cause an X-linked brain malformation phenotype with microcephaly and hypoplasia of the brainstem and cerebellum. *Nat. Genet.* 40 1065–1067 10.1038/ng.194 19165920

[B101] Nieuwenhuys R. Ten Donkelaar H. J. Nicholson C. (1998). *The Central Nervous System of Vertebrates*. Berlin: Springer-Verlag

[B102] O’Halloran C. J. Kinsella G. J. Storey E. (2012). The cerebellum and neuropsychological functioning: a critical review. *J. Clin. Exp. Neuropsychol.* 34 35–56 10.1080/13803395.2011.614599 22047489

[B103] Orvis G. D. Hartzell A. L. Smith J. B. Barraza L. H. Wilson S. L. Szulc K. U. (2012). The engrailed homeobox genes are required in multiple cell lineages to coordinate sequential formation of fissures and growth of the cerebellum. *Dev. Biol.* 367 25–39 10.1016/j.ydbio.2012.04.018 22564796 PMC4038292

[B104] Ozcelik T. Akarsu N. Uz E. Caglayan S. Gulsuner S. Onat O. E. (2008). Mutations in the very low-density lipoprotein receptor VLDLR cause cerebellar hypoplasia and quadrupedal locomotion in humans. *Proc. Natl. Acad. Sci. U.S.A.* 105 4232–4236 10.1073/pnas.0710010105 18326629 PMC2393756

[B105] Palmen S. J. Van Engeland H. Hof P. R. Schmitz C. (2004). Neuropathological findings in autism. *Brain* 127 2572–2583 10.1093/brain/awh287 15329353

[B106] Pascual M. Abasolo I. Mingorance-Le Meur A. Martinez A. Del Rio J. A. Wright C. V. (2007). Cerebellar GABAergic progenitors adopt an external granule cell-like phenotype in the absence of Ptf1a transcription factor expression. *Proc. Natl. Acad. Sci. U.S.A.* 104 5193–5198 10.1073/pnas.0605699104 17360405 PMC1829285

[B107] Pei Y. Brun S. N. Markant S. L. Lento W. Gibson P. Taketo M. M. (2012). WNT signaling increases proliferation and impairs differentiation of stem cells in the developing cerebellum. *Development* 139 1724–1733 10.1242/dev.050104 22461560 PMC3328175

[B108] Pidsley R. Dempster E. Troakes C. Al-Sarraj S. Mill J. (2012). Epigenetic and genetic variation at the IGF2/H19 imprinting control region on 11p15.5 is associated with cerebellum weight. *Epigenetics* 7 155–163 10.4161/epi.7.2.18910 22395465 PMC3335909

[B109] Ritvo E. R. Freeman B. J. Scheibel A. B. Duong T. Robinson H. Guthrie D. (1986). Lower Purkinje cell counts in the cerebella of four autistic subjects: initial findings of the UCLA-NSAC Autopsy Research Report. *Am. J. Psychiatry* 143 862–866 3717426 10.1176/ajp.143.7.862

[B110] Rodriguez C. I. Dymecki S. M. (2000). Origin of the precerebellar system. *Neuron* 27 475–486 10.1016/S0896-6273(00)00059-3 11055431

[B111] Rose M. F. Ahmad K. A. Thaller C. Zoghbi H. Y. (2009). Excitatory neurons of the proprioceptive, interoceptive, and arousal hindbrain networks share a developmental requirement for Math1. *Proc. Natl. Acad. Sci. U.S.A.* 106 22462–22467 10.1073/pnas.0911579106 20080794 PMC2799716

[B112] Sanlaville D. Verloes A. (2007). CHARGE syndrome: an update. *Eur. J. Hum. Genet.* 15 389–399 10.1038/sj.ejhg.5201778 17299439

[B113] Sato T. Joyner A. L. (2009). The duration of Fgf8 isthmic organizer expression is key to patterning different tectal-isthmo-cerebellum structures. *Development* 136 3617–3626 10.1242/dev.041210 19793884 PMC2761110

[B114] Schmahmann J. D. (1998). Dysmetria of thought: clinical consequences of cerebellar dysfunction on cognition and affect. *Trends Cogn. Sci.* 2 362–371 10.1016/S1364-6613(98)01218-2 21227233

[B115] Schmahmann J. D. (2010). The role of the cerebellum in cognition and emotion: personal reflections since 1982 on the dysmetria of thought hypothesis, and its historical evolution from theory to therapy. *Neuropsychol. Rev.* 20 236–260 10.1007/s11065-010-9142-x 20821056

[B116] Schmahmann J. D. Pandya D. N. (1989). Anatomical investigation of projections to the basis pontis from posterior parietal association cortices in rhesus monkey. *J. Comp. Neurol.* 289 53–73 10.1002/cne.902890105 2478597

[B117] Schmahmann J. D. Pandya D. N. (1991). Projections to the basis pontis from the superior temporal sulcus and superior temporal region in the rhesus monkey. *J. Comp. Neurol.* 308 224–248 10.1002/cne.903080209 1716269

[B118] Schmahmann J. D. Sherman J. C. (1998). The cerebellar cognitive affective syndrome. *Brain *121 (Pt 4) 561–579 10.1093/brain/121.4.561 9577385

[B119] Schuller U. Rowitch D. H. (2007). Beta-catenin function is required for cerebellar morphogenesis. *Brain Res.* 1140 161–169 10.1016/j.brainres.2006.05.105 16824494

[B120] Sellick G. S. Barker K. T. Stolte-Dijkstra I. Fleischmann C. Coleman R. J. Garrett C. (2004). Mutations in PTF1A cause pancreatic and cerebellar agenesis. *Nat. Genet.* 36 1301–1305 10.1038/ng1475 15543146

[B121] Selvadurai H. J. Mason J. O. (2011). Wnt/beta-catenin signalling is active in a highly dynamic pattern during development of the mouse cerebellum. *PLoS ONE* 6:e23012 10.1371/journal.pone.0023012 PMC3152553 21857982

[B122] Sgaier S. K. Lao Z. Villanueva M. P. Berenshteyn F. Stephen D. Turnbull, R. K., et al. (2007). Genetic subdivision of the tectum and cerebellum into functionally related regions based on differential sensitivity to engrailed proteins. *Development* 134 2325–2335 10.1242/dev.000620 17537797 PMC2840613

[B123] Sgaier S. K. Millet S. Villanueva M. P. Berenshteyn F. Song C. Joyner A. L. (2005). Morphogenetic and cellular movements that shape the mouse cerebellum; insights from genetic fate mapping. *Neuron* 45 27–40 10.1016/j.neuron.2004.12.021 15629700

[B124] Sherrington C. S. (1906). *The Integrative Action of the Nervous System*. New Haven: Yale University Press

[B125] Sivaswamy L. Kumar A. Rajan D. Behen M. Muzik O. Chugani D. (2010). A diffusion tensor imaging study of the cerebellar pathways in children with autism spectrum disorder. *J. Child Neurol.* 25 1223–1231 10.1177/0883073809358765 20179000

[B126] Smyser C. D. Snyder A. Z. Neil J. J. (2011). Functional connectivity MRI in infants: exploration of the functional organization of the developing brain. *Neuroimage* 56 1437–1452 10.1016/j.neuroimage.2011.02.073 21376813 PMC3089442

[B127] Spassky N. Han Y. G. Aguilar A. Strehl L. Besse L. Laclef C. (2008). Primary cilia are required for cerebellar development and Shh-dependent expansion of progenitor pool. *Dev. Biol.* 317 246–259 10.1016/j.ydbio.2008.02.026 18353302 PMC4043448

[B128] Stoodley C. J. Schmahmann J. D. (2009). Functional topography in the human cerebellum: a meta-analysis of neuroimaging studies. *Neuroimage* 44 489–501 10.1016/j.neuroimage.2008.08.039 18835452

[B129] Strick P. L. Dum R. P. Fiez J. A. (2009). Cerebellum and nonmotor function. *Annu. Rev. Neurosci.* 32 413–434 10.1146/annurev.neuro.31.060407.125606 19555291

[B130] Sudarov A. Turnbull R. K. Kim E. J. Lebel-Potter M. Guillemot F. Joyner A. L. (2011). Ascl1 genetics reveals insights into cerebellum local circuit assembly. *J. Neurosci.* 31 11055–11069 10.1523/JNEUROSCI.0479-11.2011 21795554 PMC3153985

[B131] Tavano A. Grasso R. Gagliardi C. Triulzi F. Bresolin N. Fabbro F. (2007). Disorders of cognitive and affective development in cerebellar malformations. *Brain* 130 2646–2660 10.1093/brain/awm201 17872929

[B132] Thomas K. R. Capecchi M. R. (1990). Targeted disruption of the murine int-1 proto-oncogene resulting in severe abnormalities in midbrain and cerebellar development. *Nature* 346 847–850 10.1038/346847a0 2202907

[B133] Thomas K. R. Musci T. S. Neumann P. E. Capecchi M. R. (1991). Swaying is a mutant allele of the proto-oncogene Wnt-1. *Cell* 67 969–976 10.1016/0092-8674(91)90369-A 1835670

[B134] Tohyama J. Kato M. Kawasaki S. Harada N. Kawara H. Matsui T. (2011). Dandy–Walker malformation associated with heterozygous ZIC1 and ZIC4 deletion: report of a new patient. *Am. J. Med. Genet. A* 155A 130–133 10.1002/ajmg.a.33652 21204220

[B135] Tong K. K. Kwan K. M. (2013). Common partner Smad-independent canonical bone morphogenetic protein signaling in the specification process of the anterior rhombic lip during cerebellum development. *Mol. Cell. Biol.* 33 1925–1937 10.1128/MCB.01143-12 23459943 PMC3647968

[B136] Trokovic R. Trokovic N. Hernesniemi S. Pirvola U. Vogt Weisenhorn D. M. Rossant J. (2003). FGFR1 is independently required in both developing mid- and hindbrain for sustained response to isthmic signals. *EMBO J.* 22 1811–1823 10.1093/emboj/cdg169 12682014 PMC154461

[B137] Trommsdorff M. Gotthardt M. Hiesberger T. Shelton J. Stockinger W. Nimpf J. (1999). Reeler/Disabled-like disruption of neuronal migration in knockout mice lacking the VLDL receptor and ApoE receptor 2. *Cell* 97 689–701 10.1016/S0092-8674(00)80782-5 10380922

[B138] Tsai P. T. Hull C. Chu Y. Greene-Colozzi E. Sadowski A. R. Leech J. M. (2012). Autistic-like behaviour and cerebellar dysfunction in Purkinje cell Tsc1 mutant mice. *Nature* 488 647–651 10.1038/nature11310 22763451 PMC3615424

[B139] Ueno H. Yamaguchi H. Katakami H. Matsukura S. (2004). A case of Kallmann syndrome associated with Dandy–Walker malformation. *Exp. Clin. Endocrinol. Diabetes* 112 62–67 10.1055/s-2004-815728 14758574

[B140] Vaidya C. J. (2012). Neurodevelopmental abnormalities in ADHD. *Curr. Top Behav. Neurosci.* 9 49–66 10.1007/7854_2011_138 21541845 PMC3329889

[B141] Valente E. M. Brancati F. Boltshauser E. Dallapiccola B. (2013). Clinical utility gene card for: Joubert syndrome – update 2013. *Eur. J. Hum. Genet.* 10.1038/ejhg.2013.10 [Epub ahead of print] PMC3778348 23403901

[B142] Valente E. M. Logan C. V. Mougou-Zerelli S. Lee J. H. Silhavy J. L. Brancati F. (2010). Mutations in TMEM216 perturb ciliogenesis and cause Joubert, Meckel and related syndromes. *Nat. Genet.* 42 619–625 10.1038/ng.594 20512146 PMC2894012

[B143] Villanueva R. (2012). The cerebellum and neuropsychiatric disorders. *Psychiatry Res.* 198 527–532 10.1016/j.psychres.2012.02.023 22436353

[B144] Wallace V. A. (1999). Purkinje-cell-derived Sonic hedgehog regulates granule neuron precursor cell proliferation in the developing mouse cerebellum. *Curr. Biol.* 9 445–448 10.1016/S0960-9822(99)80195-X 10226030

[B145] Wan J. Yourshaw M. Mamsa H. Rudnik-Schoneborn S. Menezes M. P. Hong J. E. (2012). Mutations in the RNA exosome component gene EXOSC3 cause pontocerebellar hypoplasia and spinal motor neuron degeneration. *Nat. Genet.* 44 704–708 10.1038/ng.2254 22544365 PMC3366034

[B146] Wang V. Y. Rose M. F. Zoghbi H. Y. (2005). Math1 expression redefines the rhombic lip derivatives and reveals novel lineages within the brainstem and cerebellum. *Neuron* 48 31–43 10.1016/j.neuron.2005.08.024 16202707

[B147] Waters S. T. Lewandoski M. (2006). A threshold requirement for Gbx2 levels in hindbrain development. *Development* 133 1991–2000 10.1242/dev.02364 16651541

[B148] Wechsler-Reya R. J. Scott M. P. (1999). Control of neuronal precursor proliferation in the cerebellum by Sonic Hedgehog. *Neuron* 22 103–114 10.1016/S0896-6273(00)80682-0 10027293

[B149] Whitty P. F. Owoeye O. Waddington J. L. (2009). Neurological signs and involuntary movements in schizophrenia: intrinsic to and informative on systems pathobiology. *Schizophr. Bull.* 35 415–424 10.1093/schbul/sbn126 18791074 PMC2659305

[B150] Wilkinson D. G. Bailes J. A. Mcmahon A. P. (1987). Expression of the proto-oncogene int-1 is restricted to specific neural cells in the developing mouse embryo. *Cell* 50 79–88 10.1016/0092-8674(87)90664-7 3594565

[B151] Wilson L. Maden M. (2005). The mechanisms of dorsoventral patterning in the vertebrate neural tube. *Dev. Biol.* 282 1–13 10.1016/j.ydbio.2005.02.027 15936325

[B152] Wingate R. (2005). Math-Map(ic)s. *Neuron* 48 1–4 10.1016/j.neuron.2005.09.012 16202701

[B153] Wingate R. J. Hatten M. E. (1999). The role of the rhombic lip in avian cerebellum development. *Development* 126 4395–4404 10498676 10.1242/dev.126.20.4395

[B154] Wittmann D. M. Blochl F. Trumbach D. Wurst W. Prakash N. Theis F. J. (2009). Spatial analysis of expression patterns predicts genetic interactions at the mid-hindbrain boundary. *PLoS Comput. Biol. * 5:e1000569 10.1371/journal.pcbi.1000569 PMC2774268 19936059

[B155] Wurst W. Auerbach A. B. Joyner A. L. (1994). Multiple developmental defects in Engrailed-1 mutant mice: an early mid-hindbrain deletion and patterning defects in forelimbs and sternum. *Development* 120 2065–2075 7925010 10.1242/dev.120.7.2065

[B156] Yang H. Jensen P. Goldowitz D. (2002). The community effect and Purkinje cell migration in the cerebellar cortex: analysis of scrambler chimeric mice. *J. Neurosci.* 22 464–470 11784791 10.1523/JNEUROSCI.22-02-00464.2002 PMC6758652

[B157] Yu T. Yaguchi Y. Echevarria D. Martinez S. Basson M. A. (2011). Sprouty genes prevent excessive FGF signalling in multiple cell types throughout development of the cerebellum. *Development* 138 2957–2968 10.1242/dev.063784 21693512 PMC3119305

